# Genetic Variants in HSP70 Family Genes and Their Co-Chaperones Are Associated with Ischemic Stroke Susceptibility and Gene Expression During Acute and Early Recovery Phases

**DOI:** 10.3390/ijms27219560

**Published:** 2026-10-26

**Authors:** Maxim Ivenkov, Anastasiya Snezhkina, Irina Shilenok, Nataliya Medvedenko, Aleksei Zarubin, Egor Bushuev, Ulyana Dubinina, Ksenia Kobzeva, Anna Kudryavtseva, Yury Nechipurenko, Olga Bushueva

**Affiliations:** 1 Research Institute for Genetic and Molecular Epidemiology, Kursk State Medical University, 305041 Kursk, Russia; 2 Department of Neurology and Neurosurgery, Kursk State Medical University, 305041 Kursk, Russia; 3 Engelhardt Institute of Molecular Biology, Russian Academy of Sciences, 119991 Moscow, Russianech99@mail.ru (Y.N.); 4 Research Institute of Medical Genetics, Tomsk National Research Medical Center, Russian Academy of Sciences, 634050 Tomsk, Russia; 5 Department of Biology, Medical Genetics and Ecology, Kursk State Medical University, 305041 Kursk, Russia; 6 Research Institute for Brain Development and Peak Performance, Peoples’ Friendship University of Russia, 117198 Moscow, Russia

**Keywords:** ischemic stroke, HSP70, heat shock proteins, BAG co-chaperones, genetic polymorphisms, single-nucleotide polymorphisms, eQTL, gene expression

## Abstract

Heat shock protein 70 (HSP70) family members and co-chaperones participate in cellular stress responses, proteostasis, apoptosis, inflammation, and mitochondrial function implicated in ischemic stroke (IS). We investigated associations between genetic variants in HSP70 family genes and BAG co-chaperones and IS susceptibility, clinical characteristics, and gene expression. Twenty-three single-nucleotide polymorphisms were analyzed in 768 patients with IS and 794 controls. Functional annotation included eQTL resources, protein–protein interaction analyses, and targeted genotype–expression analysis of paired peripheral-blood transcriptomes from 19 patients during acute IS and on day 7. In the overall cohort, the variants rs2227956 in *HSPA1L* and rs7749960 variant in the *BAG2* gene were associated with reduced IS risk. The variants rs2227956 and rs2075799 in *HSPA1L*, rs753856 in *HSPA6*, rs706121 in *BAG1*, rs7749960 in *BAG2*, and rs196329 in *BAG3* were linked to IS risk, associations specific to certain subgroups. Additional variants were associated with age at IS onset, lipid levels, coagulation parameters, body mass index, and number of IS episodes. Functional analyses linked associated loci to pathways relevant to atherosclerosis, inflammation, apoptosis, oxidative stress, autophagy, mitochondrial dysfunction, and angiogenesis. Transcriptomic analysis identified associations of rs1043618 *HSPA1A* with *C4B* expression during acute IS and *DDR1* expression on day 7, while rs1042665 *HSPA9* was associated with genotype-dependent changes in *HSPA9* expression. These findings support a role for HSP70-related genetic variation in IS susceptibility, clinical heterogeneity, and phase-specific gene-expression differences.

## 1. Introduction

Ischemic stroke (IS) remains a major medical and socioeconomic challenge and is a leading cause of mortality and long-term disability worldwide [1,2]. Its pathogenesis involves a complex cascade of pathophysiological processes, including excitotoxicity [3], oxidative stress [4,5,6], mitochondrial dysfunction [7], and apoptosis [8,9]. Triggered by a critical reduction in cerebral blood flow, these processes ultimately result in extensive neuronal injury and death [10]. Atherosclerotic vascular disease is a major contributor to ischemic stroke, highlighting the relevance of molecular mechanisms involved in vascular homeostasis and atherogenesis [11,12].

Disruption of cellular proteostasis plays an important role in both ischemic neuronal injury and the mechanisms underlying resistance to ischemic damage [13,14,15]. A central component of proteostasis is the protein quality control system, in which molecular chaperones (MCs) play a key role. In ischemic stroke, MCs contribute to neuroprotection by preventing the aggregation of proteins denatured under conditions of ischemic and hypoxic stress and by facilitating both the folding of newly synthesized polypeptides and the refolding of damaged proteins [16,17]. Molecular chaperones are also implicated in several processes relevant to atherosclerosis, including metabolic regulation [18], redox homeostasis [19], endothelial dysfunction [20], vascular smooth muscle cell proliferation [21], inflammation [22], and autophagy [23].

Among molecular chaperones, the heat shock protein 70 (HSP70) family is of particular interest because of its important role in cellular stress responses and vascular homeostasis [24,25]. HSP70 proteins modulate inflammatory responses and endothelial function through several mechanisms, including regulation of NF-κB activation [26], endothelial nitric oxide synthase activity [27], cytokine secretion [28], and apoptosis [25]. These functions may contribute to their involvement in atherosclerosis and other vascular processes associated with ischemic stroke. HSP70 proteins also promote cell survival under conditions of hypoxia, ischemia, and oxidative stress [16]. Following cerebral ischemia, HSP70 expression is strongly induced in response to the accumulation of misfolded and damaged proteins, particularly in neurons within the ischemic penumbra [29]. HSP70-mediated neuroprotection has been attributed to the stabilization and refolding of damaged proteins [30], inhibition of apoptosis [31], and attenuation of neuroinflammation [32]. Increased HSP70 expression following reperfusion has also been associated with reduced brain lesion volume and improved neurological outcomes [33].

The HSP70 family comprises multiple members with distinct but partially overlapping functions. Among them, HSPA1L, HSPA4, and HSPA6 together with the HSP70-associated co-chaperones BAG1, BAG2, and BAG3, are of particular interest in the context of ischemic stroke. These proteins participate in processes relevant to ischemic brain injury and vascular pathology, including maintenance of vascular homeostasis [27], regulation of inflammatory responses [34] and apoptosis [35], protection against oxidative stress [36], and maintenance of mitochondrial homeostasis [37]. Collectively, these functions support their potential involvement in determining susceptibility to ischemic injury and its clinical consequences [38,39,40]. However, despite substantial molecular and experimental evidence implicating HSP70 proteins in ischemic stroke and ischemic brain injury, the contribution of genetic variation in HSP70 family genes and their co-chaperones remains poorly characterized.

This study presents a comprehensive analysis genetic variants in genes encoding selected HSP70 family proteins and their co-chaperones in relation to IS susceptibility and clinical characteristics. Given the multifactorial nature of IS and the contribution of both genetic and environmental factors to disease risk, particular attention was also given to potential gene–environment interactions. In addition, we used a range of bioinformatic approaches to investigate the potential functional consequences of the identified variants and to elucidate molecular mechanisms through which these genes may contribute to IS development and clinical manifestations.

## 2. Results

### 2.1. Associations Between HSP70-Related SNPs and Ischemic Stroke Risk

This study involved 1 562 unrelated residents of Central Russia, comprising 768 patients with IS and 794 control participants. The analysis included the *HSPA1A*, *HSPA1L*, *HSPA2*, *HSPA4*, *HSPA5*, *HSPA6*, *HSPA9*, and *HSPA14* genes, encoding members of the HSP70 family, as well as *BAG1*, *BAG2*, *BAG3*, *BAG4*, and *BAG5*, encoding HSP70-associated co-chaperones.

**Figure 1:**
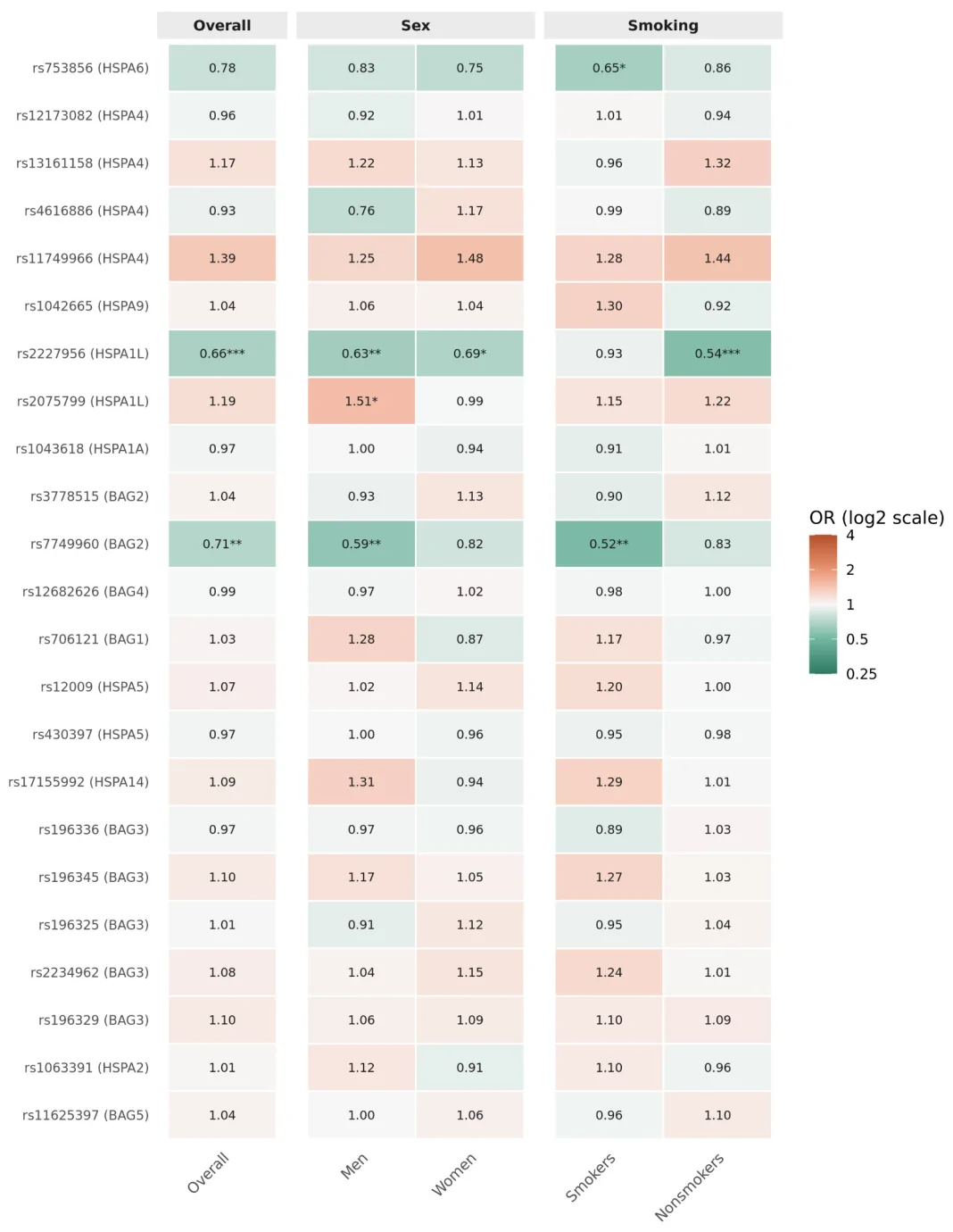
Heatmap of *HSP70*-related genetic associations with ischemic stroke risk (overall, sex, smoking). The heatmap summarizes associations between 23 SNPs in *HSP70* family and co-chaperone genes and IS risk in the overall study population and in subgroups stratified by sex (men and women) and smoking status (smokers and nonsmokers). Associations were assessed using logistic regression under an additive genetic model, with adjustment for age, sex, and smoking status, as applicable. Cell colors represent the log_2_-transformed odds ratios (ORs), while numerical values indicate the corresponding ORs for the effect alleles. Asterisks indicate statistical significance: *p* < 0.05 (*), *p* < 0.01 (**), and *p* < 0.001 (***). OR > 1 indicates an association with increased IS risk, whereas OR < 1 indicates an association with reduced risk.

For an integrated overview of the genetic associations across the overall cohort and subgroup analyses, see Figure 1. In the overall sample, the G allele of rs2227956 *HSPA1L* was associated with a reduced risk of IS (OR = 0.66, 95% CI 0.53–0.83, *p_perm_* = 0.0004), as was the A allele of rs7749960 *BAG2* (OR = 0.72, 95% CI 0.56–0.92, *p_perm_* = 0.01) (Table 1).

Pairwise LD analysis revealed low-to-moderate correlations between rs2227956 and rs2075799 in *HSPA1L* and rs1043618 in *HSPA1A* (r^2^ = 0.028–0.214), despite high D′ values (0.919–1.000). The investigated *HSPA4* variants showed generally low pairwise r^2^ values (≤0.05). In contrast, several *BAG3* variants exhibited moderate pairwise correlations (r^2^ up to 0.462; D′ up to 0.994), indicating that some of these variants are not statistically independent. Complete pairwise LD statistics are presented in Table S1, with a graphical summary provided in Figure S1.

Associations identified in subgroups stratified by sex, smoking status, physical activity, and fruit and vegetable intake are presented in Tables S2–S4. Sex-stratified analysis revealed several sex-specific patterns. In men, the G allele of rs2227956 *HSPA1L* (OR = 0.63, 95% CI 0.47–0.86, *p_perm_* = 0.003) and the A allele of rs7749960 *BAG2* (OR = 0.58, 95% CI 0.41–0.82, *p_perm_* = 0.002) were associated with a reduced risk of IS, whereas the T allele of rs2075799 *HSPA1L* (OR = 1.53, 95% CI 1.08–2.16, *p_perm_* = 0.02) and the C allele of rs706121 *BAG1* (OR = 1.40, 95% CI 1.04–1.89, *p_perm_* = 0.02) were associated with an increased risk. In women, the G allele of rs753856 *HSPA6* was associated with a reduced risk of IS (OR = 0.70, 95% CI 0.50–0.98, *p_perm_* = 0.03) (Table 2).

Further analyses were performed in subgroups stratified by major cardiovascular and cerebrovascular risk factors, including smoking, physical activity, and fruit and vegetable intake. Among smokers, the A allele of rs7749960 *BAG2* was associated with a reduced risk of IS (OR = 0.50, 95% CI 0.34–0.74, *p_perm_* = 0.001). Among non-smokers, the G allele of rs2227956 *HSPA1L* was associated with a reduced risk of IS (OR = 0.60, 95% CI 0.45–0.79, *p_perm_* = 0.001) (Table 3).

In exploratory analyses comparing IS patient subgroups with the entire control group, several associations were identified according to physical activity and fruit and vegetable intake (Table 4). Among patients reporting low physical activity, the G allele of rs2227956 (*HSPA1L*; OR = 0.66, 95% CI 0.49–0.88, *p_perm_* = 0.005, *p_bonf_* = 0.01) and the A allele of rs7749960 (*BAG2*; OR = 0.69, 95% CI 0.50–0.95, *p_perm_* = 0.02, *p_bonf_* = 0.04) were associated with lower odds of IS relative to controls. In contrast, among patients reporting normal physical activity, a reduced odds of IS was associated with the G allele of rs753856 (*HSPA6*; OR = 0.60, 95% CI 0.40–0.91, *p_perm_* = 0.02, *p_bonf_* = 0.04).

In analyses stratified by fruit and vegetable intake, the G allele of rs753856 (*HSPA6*; OR = 0.69, 95% CI 0.51–0.93, *p_perm_* = 0.02, *p_bonf_* = 0.04) and the A allele of rs7749960 (*BAG2*; OR = 0.62, 95% CI 0.47–0.84, *p_perm_* = 0.001, *p_bonf_* = 0.002) were associated with reduced IS odds when patients with low intake were compared with controls. Among patients reporting normal intake, the G allele of rs2227956 (*HSPA1L*; OR = 0.69, 95% CI 0.53–0.90, *p_perm_* = 0.005, *p_bonf_* = 0.01) was associated with lower IS odds, whereas the A allele of rs196329 (*BAG3*; OR = 1.25, 95% CI 1.03–1.52, *p_perm_* = 0.02, *p_bonf_* = 0.04) was associated with higher odds.

Formal heterogeneity analyses were performed to assess whether the associations between the investigated SNPs and IS risk differed by sex or smoking status (Tables S5 and S6). No SNP showed nominally significant heterogeneity between men and women (minimum *p* = 0.051). In particular, the apparent sex differences observed for rs706121 *BAG1* and rs2075799 *HSPA1L* were not supported by formal heterogeneity testing (*p* = 0.082 and *p* = 0.087, respectively). In the smoking-stratified analysis, rs2227956 *HSPA1L* showed nominal evidence of heterogeneity (*p* = 0.029), which did not remain significant after FDR correction (q = 0.67). Overall, none of the investigated variants demonstrated statistically significant heterogeneity by sex or smoking status after correction for multiple testing.

### 2.2. Associations of HSP70-Related SNPs with Clinical and Biochemical Characteristics of Patients with Ischemic Stroke

**Figure 2:**
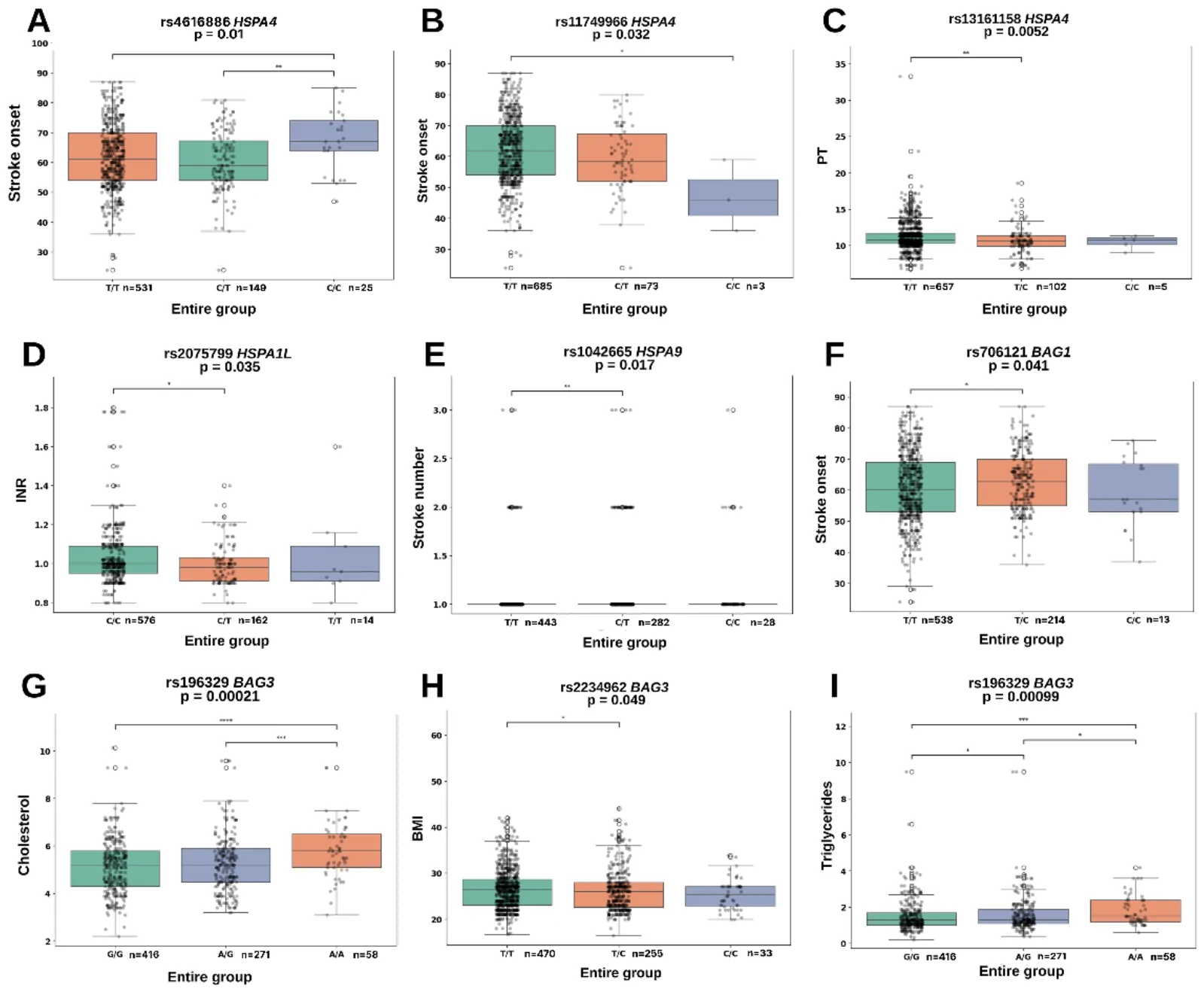
Associations of *HSP70* SNPs and clinical parameter in the entire group. (**A**)—stroke onset data for rs4616886 *HSPA4*, (**B**)—stroke onset data for rs11749966 *HSPA4*, (**C**)—PT values for rs13161158 *HSPA4*, (**D**)—INR values for rs2075799 *HSPA1L*, (**E**)—stroke number data for rs1042665 *HSPA9*, (**F**)—stroke onset data for rs706121 *BAG1*, (**G**)—cholesterol values for rs196329 *BAG3*, (**H**)—BMI values for rs2234962 *BAG3*, (**I**)—triglycerides values for rs196329 *BAG3*; The number of stars indicates the level of significance of the differences: *—*p* < 0.05; **—*p* < 0.01; ***—*p* < 0.001; ****—*p* < 0.0001.

To further characterize the clinical relevance of HSP70-related variants, we examined their associations with age at IS onset, number of IS episodes, body mass index (BMI), blood lipid parameters, including total cholesterol and triglyceride levels, and coagulation parameters, including the international normalized ratio (INR) and prothrombin time (PT), among patients with IS (Figure 2).

Two *HSPA4* variants, rs4616886 (*p* = 0.013) and rs11749966 (*p* = 0.032), were associated with age at IS onset. In addition, rs13161158 *HSPA4* was associated with PT (*p* = 0.0052), whereas the *HSPA1L* variant rs2075799 was associated with INR (*p* = 0.035). The *HSPA9* variant rs1042665 was associated with the number of IS episodes (*p* = 0.017). The *BAG1* variant rs706121 was also associated with age at IS onset, while the *BAG2* variant rs3778515 was associated with BMI (*p* = 0.018). Among the investigated *BAG3* variants, rs2234962 was associated with BMI (*p* = 0.049), whereas rs196329 was associated with both triglyceride (*p* = 0.00099) and total cholesterol levels (*p* = 0.00021).

**Figure 3:**
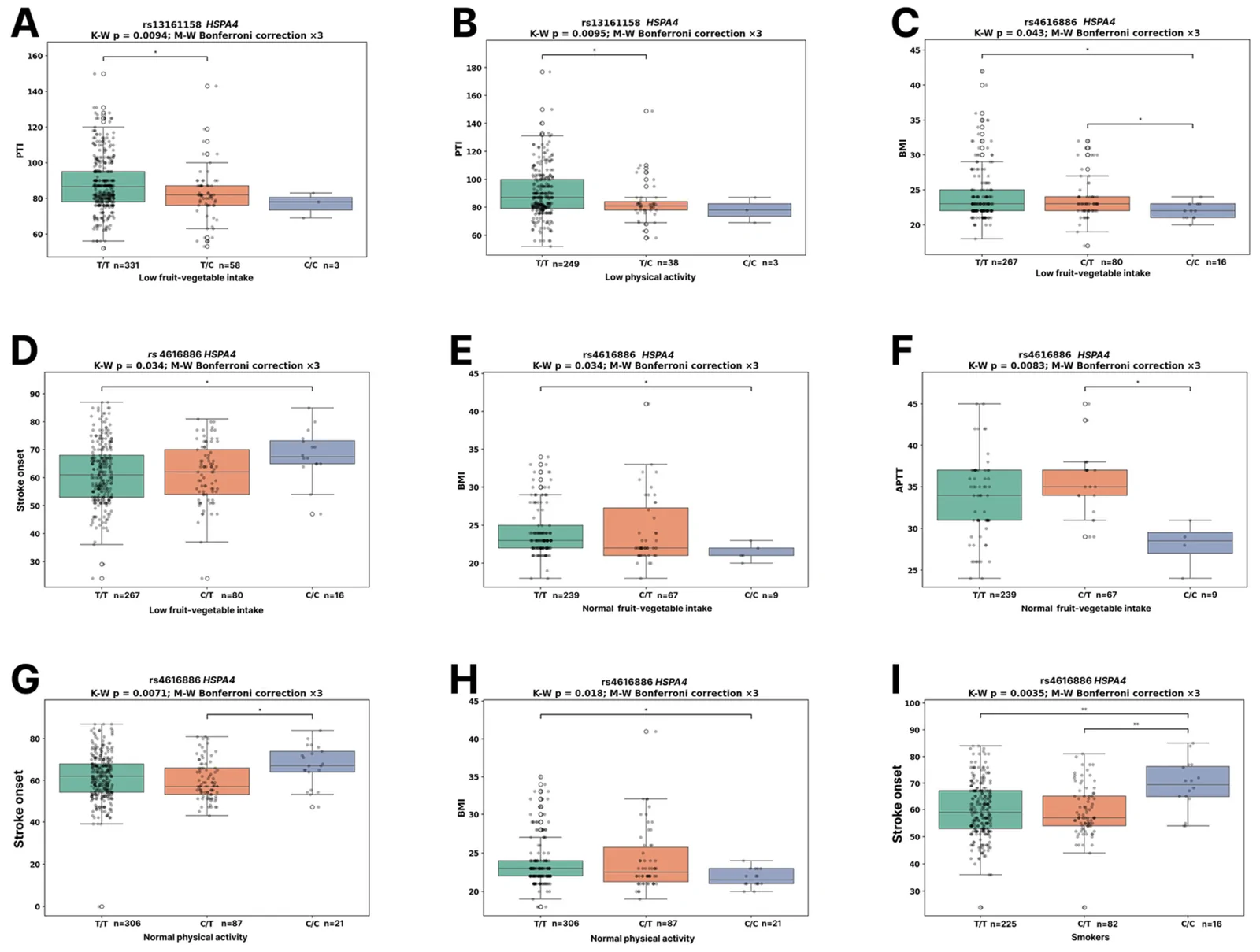
Associations between *HSP70*-related SNPs and clinical characteristics in patient subgroups. (**A**) Prothrombin index according to rs13161158 *HSPA4* genotype in patients with low fruit and vegetable intake; (**B**) prothrombin index according to rs13161158 *HSPA4* genotype in patients with low physical activity; (**C**) body mass index according to rs4616886 *HSPA4* genotype in patients with low fruit and vegetable intake; (**D**) age at stroke onset according to rs4616886 *HSPA4* genotype in patients with low fruit and vegetable intake; (**E**) body mass index according to rs4616886 *HSPA4* genotype in patients with normal fruit and vegetable intake; (**F**) activated partial thromboplastin time (aPTT) according to rs4616886 *HSPA4* genotype in patients with normal fruit and vegetable intake; (**G**) age at stroke onset according to rs4616886 *HSPA4* genotype in patients with normal physical activity; (**H**) body mass index according to rs4616886 *HSPA4* genotype in patients with normal physical activity; (**I**) age at stroke onset according to rs4616886 *HSPA4* genotype in smokers; The number of stars indicates the level of significance of the differences: *—*p* < 0.05; **—*p* < 0.01.

Subgroup analyses stratified by lifestyle-related risk factors identified additional associations involving rs13161158 and rs4616886 *HSPA4* (Figure 3). rs13161158 *HSPA4* was associated with higher prothrombin index (PTI) among patients with low physical activity (*p* = 0.0095) and those with low fruit and vegetable intake (*p* = 0.0094). rs4616886 *HSPA4* was associated with several clinical parameters across the analyzed subgroups. Among patients with low fruit and vegetable intake, the variant was associated with BMI (*p* = 0.043) and age at IS onset (*p* = 0.034). Among those with normal fruit and vegetable intake, it was associated with BMI (*p* = 0.034) and activated partial thromboplastin time (aPTT; *p* = 0.0083). In patients with normal physical activity, rs4616886 *HSPA4* was associated with age at IS onset (*p* = 0.0071) and BMI (*p* = 0.018), while among smokers it was associated with age at IS onset (*p* = 0.0035).

### 2.3. PPI-Analysis

**Figure 4:**
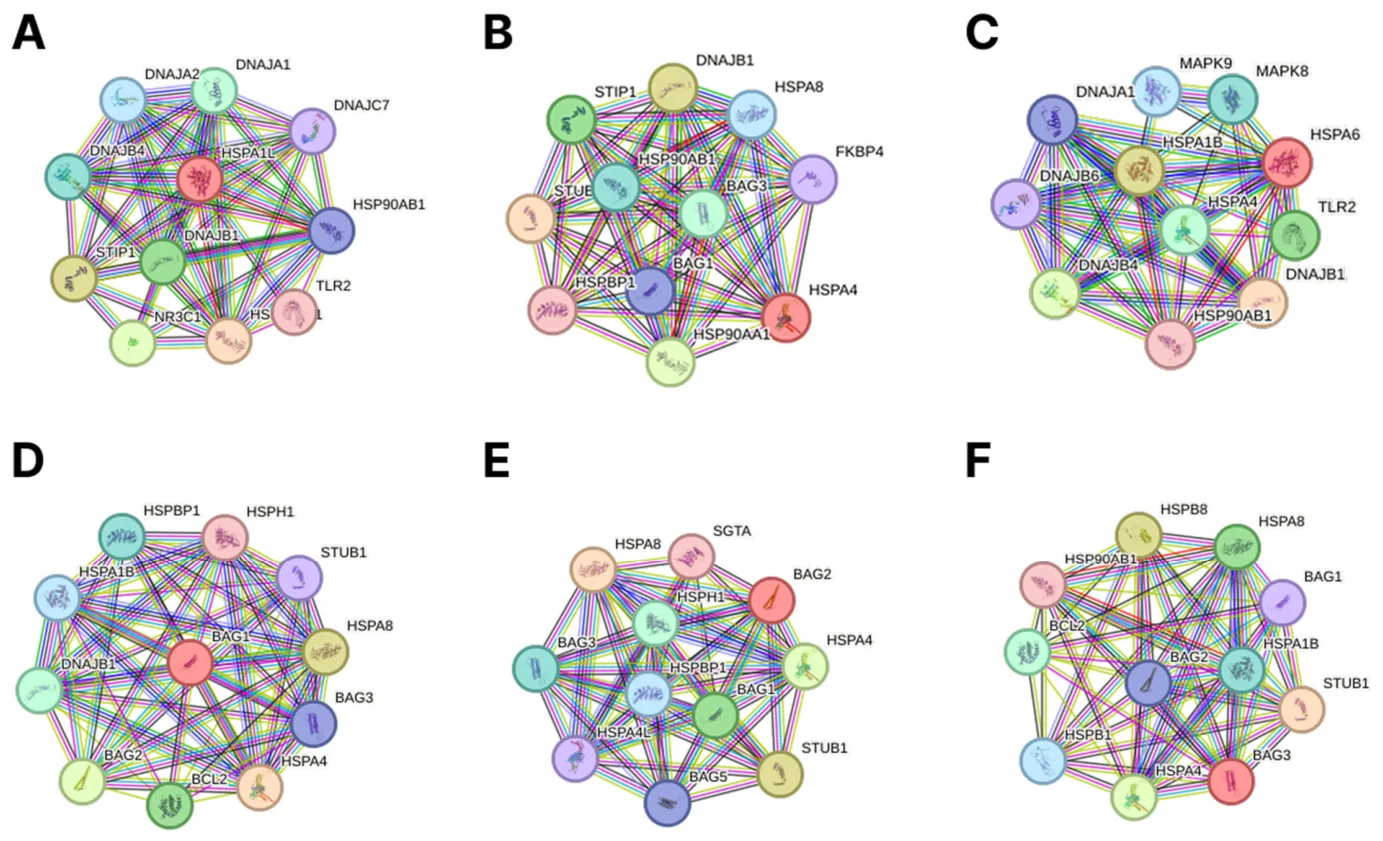
Protein–protein-interaction networks of selected HSP70 family proteins and BAG co-chaperones generated using the STRING database. The main functional interaction partners are shown for (**A**) HSPA1L, (**B**) HSPA4, (**C**) HSPA6, (**D**) BAG1, (**E**) BAG2, and (**F**) BAG3.

STRING-based protein–protein interaction analysis identified functional networks associated with 6 proteins: HSPA1L, HSPA4, HSPA6, BAG1, BAG2, and BAG3 (Figure 4). Complete lists of the top 10 PPI partners for each of the proteins under study are presented in Figure 4 and Table S7. The resulting 60 query–partner associations included 29 distinct partner protein. Key interaction partners included proteins involved in molecular chaperoning and proteostasis maintenance (specifically HSPA8, HSP90AA1, HSP90AB1, STIP1, STUB1, HSPBP1, and several members of the DNAJ co-chaperone family), as well as proteins involved in the regulation of apoptosis and stress response signaling (including BCL2, MAPK8, MAPK9, and TLR2.

### 2.4. Functional Annotation and Genotype–Expression Associations of IS-Associated Variants

Following the genetic association analyses, we investigated the potential functional consequences of SNPs associated with IS risk using publicly available eQTL resources and regulatory annotation tools.

Analysis of eQTLGen data (Table S8) revealed extensive cis-eQTL associations in peripheral blood. The strongest pleiotropic effects were observed for the *HSPA1L* variants rs2227956 and rs2075799. rs2227956 was associated with the expression of 49 genes, including multiple genes within the major histocompatibility complex (MHC) region, such as *HLA-B*, *HLA-C*, *HLA-DQB1*, *HLA-DRB1*, *HLA-DRB5*, *C4A*, *C4B*, *MICB*, and *TNF*, as well as *HSPA1B*, *DDR1*, *DDAH2*, and *BAG6*. Similarly, rs2075799 was associated with the expression of 28 genes, including *HSPA1L*, *HLA*-*C*, *HLA*-*DQB1*, *HLA*-*DRB1*, *HLA*-*DRB5*, *C4A*, *MICB*, *DDR1*, *DDAH2*, and *CSNK2B*.

Several additional variants showed more restricted cis-eQTL effects. rs4616886 *HSPA4* was associated with expression of *SHROOM1*, *HSPA4*, *UQCRQ*, *SLC22A5*, and *LEAP2*, whereas rs13161158 *HSPA4* was associated with expression of *AFF4*, *HSPA4*, *SLC22A5*, *LEAP2*, and *SLC22A4*. The *HSPA6* variant rs753856 was associated with expression of several Fc receptor-related genes, including *FCGR2A*, *FCGR2B*, *FCGR2C*, and *FCRLB*. Additional eQTL associations were identified for rs706121 *BAG1* with *AQP3*, *NOL6*, *NFX1*, *BAG1*, and *MCMBP*; rs7749960 *BAG2* with *RAB23*, *KIAA1586*, *BAG2*, and *ZNF451*; rs2234962 with *MCMBP* and *BAG3*; and rs196329 *BAG3* with *BAG3*, *MCMBP*, *KRIT1*, *CYP51A1*, *ANKIB1*, and *AKAP9*. The complete list of eQTL associations is provided in Table S8.

GTEx analysis further demonstrated tissue-specific eQTL effects of the investigated variants in whole blood, arterial tissues, and brain tissues (Table S9). rs2227956 *HSPA1L* showed particularly extensive eQTL effects across these tissues and was associated with the expression of multiple genes within the MHC region, including *HLA-B*, *HLA-C*, *HLA-DQB1*, *HLA-DRB1*, *HLA-DRB5*, *C4A*, *C4B*, and *CFB*, as well as *HSPA1L*, *HSPA1B*, *DDAH2*, and *BAG6*. rs2075799 was associated with expression of *HSPA1L*, *STK19*, *LSM2*, *LY6G5C*, *ATP6V1G2*, and *VARS2* across whole-blood, arterial, and/or brain tissues. Other tissue-specific eQTL associations included rs4616886 with *SHROOM1*, rs7749960 with *LINC00680* and *KIAA1586*, rs706121 *BAG1* with *NFX1* and *AQP3*, and rs2234962 *BAG3* with *INPP5F*. Together, these results indicate that several IS-associated variants are linked to gene-expression differences in tissues relevant to vascular and cerebrovascular disease.

To further investigate genotype-dependent gene expression in the context of IS, we performed a targeted analysis of nine predefined SNP–gene pairs using paired whole-blood RNA-seq profiles obtained from 19 patients during the acute phase of IS and on day 7 after stroke. Three hypotheses were evaluated for each SNP–gene pair: an association between genotype and expression during the acute phase, an association between genotype and expression on day 7, and a genotype-dependent difference in expression change between the two time points. In total, 27 tests were performed. Five associations had nominal *p* < 0.05, of which three remained significant after Benjamini–Hochberg correction within the corresponding hypothesis family (FDR < 0.05).

**Figure 5:**
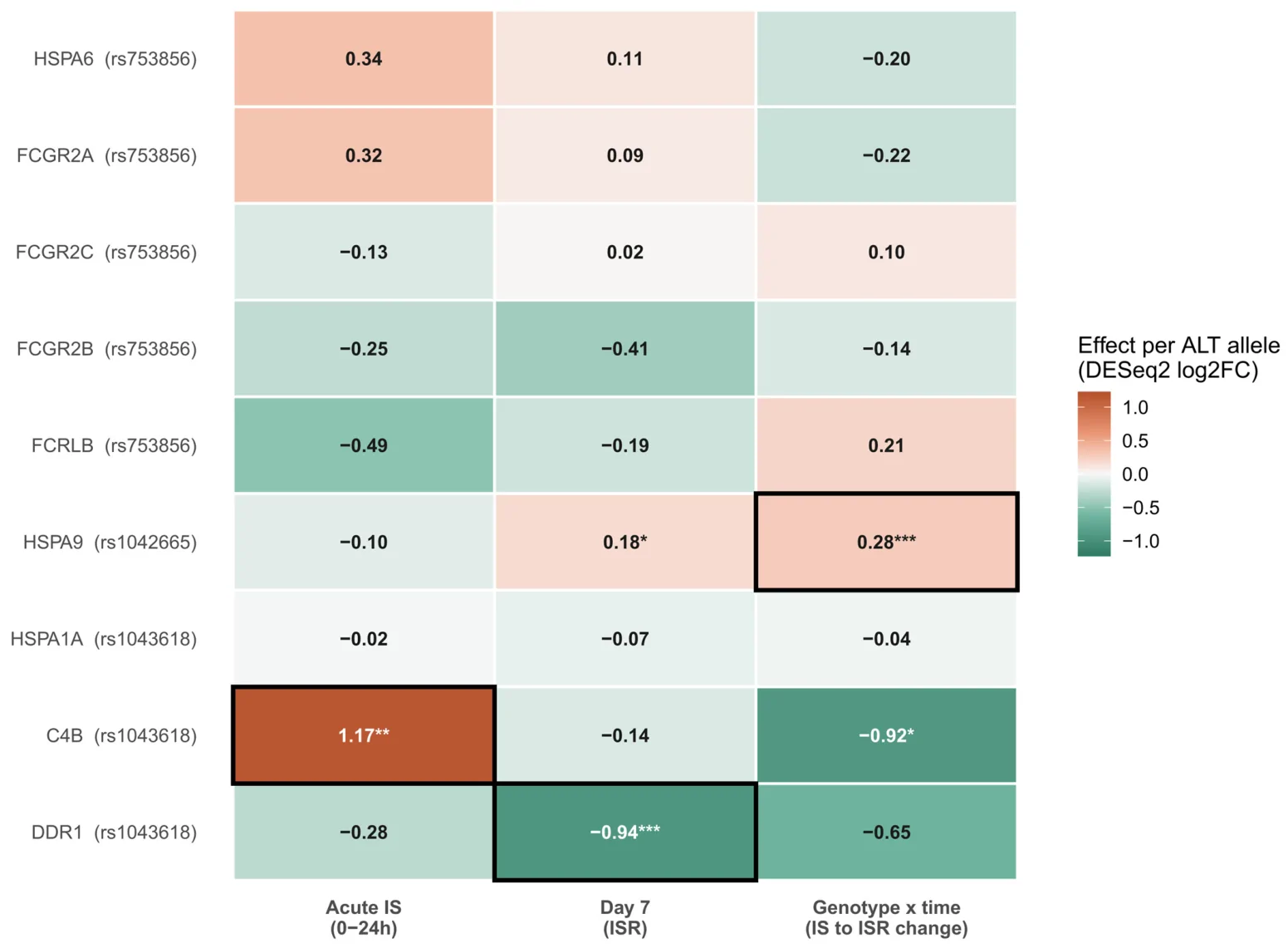
Targeted genotype–expression associations in the paired ischemic stroke RNA-seq cohort. Heatmap of targeted genotype–expression associations in paired whole-blood RNA-seq samples from 19 patients with ischemic stroke (acute phase within 24 h and day 7/ISR). Rows show the nine prioritized SNP–gene pairs for rs753856 (*HSPA6* and four eQTLGen-supported cis genes), rs1042665 (*HSPA9*), and rs1043618 (*HSPA1A*, *C4B*, and *DDR1*); columns show the three prespecified DESeq2 hypotheses: genotype effect during acute IS, genotype effect at day 7, and genotype-by-time interaction on the IS-to-ISR expression change. Cell color and value show the model-estimated effect per additional ALT allele (DESeq2 log2 fold-change). Asterisks denote nominal significance (*p* < 0.05, *p* < 0.01, *p* < 0.001); black outlines denote significance after Benjamini–Hochberg correction (FDR < 0.05) within each hypothesis family.

Figure 5 summarizes all 27 targeted genotype–expression tests (nine SNP–gene pairs across three hypotheses) as a heatmap, providing an integrated overview of the targeted analysis.

**Figure 6:**
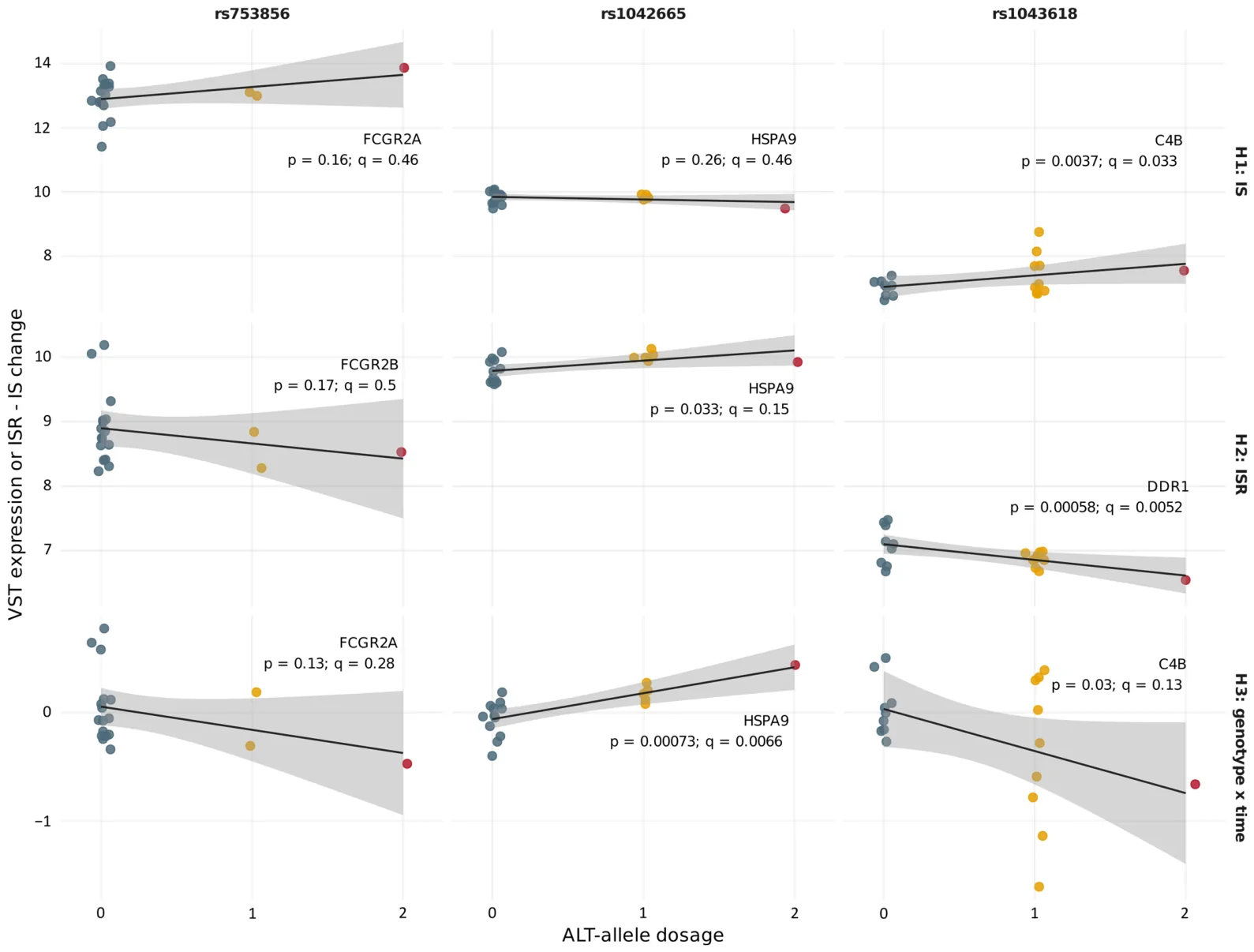
Top targeted genotype–expression association for each SNP and hypothesis in the paired ischemic stroke RNA-seq cohort. Columns correspond to rs753856, rs1042665, and rs1043618; rows correspond to the genotype effect during acute IS (H1), the genotype effect on day 7 (H2), and the genotype-by-time effect on the IS-to-ISR expression change (H3). Within each SNP–hypothesis combination, the plotted gene had the smallest primary DESeq2 *p*-value among the targeted genes for that SNP. Points represent patients and are colored by alternative-allele dosage; black lines and shaded areas show descriptive linear fits and 95% confidence intervals. The y-axis shows VST expression for H1 and H2 and paired ISR-minus-IS VST change for H3. Labels report nominal *p*-values and BH-adjusted *p*-values from the primary DESeq2 models, not from the plotted linear fits.

During the acute phase of IS, rs1043618 *HSPA1A* was positively associated with *C4B* expression (effect estimate = 1.170, *p* = 0.00371, FDR = 0.0334), indicating higher *C4B* expression with increasing dosage of the alternative allele (Figure 6). On day 7, rs1043618 was negatively associated with *DDR1* expression (effect estimate = −0.940, *p* = 0.000579, FDR = 0.00521), corresponding to lower *DDR1* expression with increasing alternative-allele dosage (Figure 6).

The strongest genotype-dependent longitudinal effect was observed for rs1042665 and *HSPA9*. The genotype-by-time effect was positive (effect estimate = 0.276, *p* = 0.000728, FDR = 0.00655), indicating that the change in *HSPA9* expression between the acute phase and day 7 differed according to rs1042665 genotype (Figure 6). Although three associations remained significant after correction for multiple testing, these findings should be interpreted as exploratory given the limited sample size of the RNA-seq cohort and the small number of individuals within certain genotype groups. Independent validation in larger cohorts is required to determine the reproducibility and biological relevance of these associations.

## 3. Discussion

To our knowledge, this study represents the first comprehensive investigation of associations between genetic variation in *HSP70* family genes and their co-chaperones and both the risk and clinical characteristics of IS. Using a large case–control cohort, we examined associations with IS susceptibility, clinical and biochemical parameters, and patterns observed across subgroups defined by major environmental and lifestyle-related risk factors. These analyses were complemented by functional bioinformatic annotation and by targeted genotype–expression analysis using paired whole-blood transcriptomic profiles obtained from patients during the acute phase of IS and on day 7 after stroke. Together, these approaches allowed us to investigate not only genetic associations with IS but also potential molecular mechanisms through which the identified variants may contribute to disease susceptibility and clinical phenotype.

Among the 23 investigated SNPs, several variants in *HSP70* family genes and their co-chaperones were associated with IS risk. These included variants in *HSPA1L*, *HSPA6*, *BAG1*, *BAG2*, and *BAG3*. Several associations differed between men and women and were also observed within subgroups stratified by smoking status, physical activity, and fruit and vegetable intake. In addition, nine variants in *HSPA1L*, *HSPA4*, *HSPA9*, *BAG1*, *BAG2*, and *BAG3* were associated with clinical or biochemical characteristics of IS, including age at disease onset, number of IS episodes, coagulation parameters, lipid levels, and BMI. Given that IS is a multifactorial disease, and these phenotypes may be influenced by numerous genetic and environmental factors, as well as disease-related changes and medical treatment, the observed associations do not establish that individual *HSP70*-related variants directly influence coagulation parameters, lipid metabolism, or other clinical characteristics.

Functional analyses further indicated that several IS-associated variants were linked to extensive differences in gene expression. In total, 95 genes showed eQTL associations with the investigated variants in publicly available datasets. Many of these genes have previously been implicated in biological processes relevant to IS, including atherosclerosis, inflammation, cell survival and apoptosis, autophagy, mitochondrial dysfunction, oxidative stress, excitotoxicity, and angiogenesis. In addition, STRING analysis identified 15 unique interaction partners of the investigated HSP70-related proteins that have also been implicated in major molecular mechanisms underlying ischemic brain injury. Targeted analysis of paired transcriptomic data from patients with IS provided further evidence of genotype-dependent expression differences, including associations involving *C4B*, *DDR1*, and *HSPA9* during the acute and subacute phases of stroke.

Previous candidate-gene studies have investigated associations between polymorphisms in *HSPA1A*, *HSPA1B*, and *HSPA1L* and cerebrovascular disease, with inconsistent findings. In a prospective study, Zee et al. (2002) found no significant association between the HSP70-hom +2437T/C polymorphism (rs2227956) and stroke risk [41]. Similarly, Liu et al. (2007) reported an association involving HSP70-1 +190G/C but not HSP70-hom +2437T/C [42]. In contrast, Wei et al. (2013) identified associations involving the +2437T/C polymorphism and ischemic stroke in a Chinese Han population and reported potential interactions with hypertension [43]. These findings demonstrate that genetic variation in the HSP70 gene family has previously been investigated in relation to cerebrovascular disease, although the available evidence remains inconsistent. Our findings therefore extend previous research by examining additional HSP70-related genes and co-chaperones alongside functional annotation and genotype–expression analyses. Nevertheless, the potential influence of population differences, linkage disequilibrium, and cardiovascular risk factors requires consideration when interpreting these associations.

The *HSPA1L* gene is involved in mitochondrial quality control and mitophagy, processes required for the maintenance of mitochondrial function and ATP production and therefore of particular importance in energy-demanding tissues such as the brain [37]. Furthermore, an association between the *HSPA1L* SNP rs2227956 (risk allele G) and CCL19 (C-C motif chemokine 19) protein levels was identified in the GWAS Catalog (NHGRI-EBI). Data from study GCST90088816 indicate that, in a European population (n = 5367), this SNP shows a statistically significant association with CCL19 levels (β = 0.167141 unit increase, *p* = 7 × 10^−14^) [44]. CCL19 is of interest as a pro-inflammatory chemokine that potentially links immune activation to the progression and destabilization of atherosclerotic plaques [45]. In our study, rs2227956 in *HSPA1L* was associated with IS risk in the overall cohort and in several subgroups defined by sex and lifestyle-related risk factors. Another *HSPA1L* variant, rs2075799, was associated with increased IS risk in men. rs2075799 was also associated with INR among patients with IS. The T allele was associated with lower INR values, suggesting a potential relationship between this variant and coagulation status. Given the importance of coagulation abnormalities and thrombotic mechanisms in IS [46], this association warrants further investigation.

The *HSPA1L* variants rs2227956 and rs2075799 showed extensive eQTL effects and were collectively associated with the expression of 58 genes. Importantly, several of these genes have previously been implicated in molecular processes relevant to atherosclerosis and cerebral ischemia, including *POU5F1*, *DDR1*, *DDAH2*, *TNF*, *NOTCH4*, *EHMT2*, *TNXB*, *BAG6*, *VARS2*, *HLA*-*DRB1*, *HLA*-*DQB1*, *RNF5*, *ATF6B*, and *HSPA1L*. These observations suggest that the associations between *HSPA1L* variants and IS may extend beyond effects on *HSPA1L* itself and involve broader regulatory networks related to vascular dysfunction, inflammation, cellular stress responses, and mitochondrial homeostasis.

Several of the identified eQTL genes are involved in vascular pathology and atherogenesis. *POU5F1*, whose expression was associated with both rs2227956 and rs2075799, has been implicated in recovery following cerebral ischemia; increased *POU5F1* expression in the brains of mice subjected to cerebral ischemia was associated with accelerated functional recovery [47]. *DDR1*, another eQTL target shared by the two *HSPA1L* variants, has been linked to increased blood–brain barrier permeability and infarct volume, partly through enhanced expression and activity of matrix metalloproteinase-9 (MMP-9) [48]. *DDR1* also contributes to multiple stages of atherogenesis by promoting macrophage infiltration into the collagen-rich extracellular matrix of the arterial wall [49]. *DDAH2* is involved in the maintenance of endothelial nitric oxide homeostasis, whereas reduced *DDAH2* expression has been associated with impaired NO signaling and increased endothelial cell apoptosis [50]. In addition, *TNF* represents a plausible link between inflammation and vascular pathology: elevated TNF levels have been associated with the risk of both first and recurrent stroke [51], while TNF-family signaling has also been implicated in atherosclerosis [52]. Sustained TNF signaling may additionally impair angiogenesis through interference with VEGF signaling [53].

Other eQTL targets further support a potential connection with endothelial dysfunction and atherosclerosis. *NOTCH4* is highly relevant in this context because JAG1–NOTCH4 signaling is activated in endothelial cells exposed to disturbed blood flow at atherosclerosis-prone vascular sites. Activation of this pathway promotes atherosclerosis susceptibility by altering endothelial proliferative and migratory responses [54]. Reduced expression of *TNXB* has likewise been associated with accelerated atherosclerosis development [55]. Together, these findings suggest that the broad eQTL effects of the *HSPA1L* variants may influence several pathways involved in vascular homeostasis and atherogenesis.

A second group of eQTL-associated genes is involved in cellular stress responses and mechanisms directly relevant to ischemic brain injury. Inhibition of *EHMT2* has been shown to protect cells in the ischemic penumbra from apoptosis and reduce cerebral infarct volume, supporting pro-apoptotic and pro-inflammatory functions of *EHMT2* during cerebral ischemia [56]. Increased *BAG6* expression has been reported to attenuate ischemic brain injury by reducing oxidative stress, preserving mitochondrial function, and modulating autophagy [57]. *VARS2*, a mitochondrial aminoacyl-tRNA synthetase, is essential for mitochondrial protein synthesis and consequently for respiratory-chain function and ATP production; impairment of *VARS2* therefore particularly affects tissues with high energetic demands, including the brain [58]. The identified associations with *HLA-DRB1* and *HLA-DQB1* additionally point to potential involvement of immune-regulatory mechanisms, as variation within these genes has been linked to susceptibility to autoimmune and inflammatory responses [59].

Finally, several eQTL targets are directly involved in protein quality control, autophagy, and mitochondrial homeostasis, providing a mechanistic link to the established functions of the HSP70 family. *RNF5* promotes ubiquitination and degradation of ATG4B, an important regulator of autophagy [60], whereas *ATF6B* participates in the endoplasmic-reticulum stress response to the accumulation of misfolded proteins [61]. *HSPA1L* itself has been implicated in mitochondrial quality control by facilitating recruitment of Parkin to damaged mitochondria [37]. Following activation by the mitochondrial damage sensor PINK1, Parkin accumulates on the outer mitochondrial membrane and promotes ubiquitination-dependent removal of damaged mitochondria [62,63]. The coordinated PINK1–Parkin pathway therefore contributes to mitochondrial quality control and cell survival under stress conditions [64]. Collectively, these eQTL findings support the possibility that *HSPA1L* variants associated with IS may exert their effects through coordinated regulation of vascular, inflammatory, proteostatic, and mitochondrial pathways rather than through changes in *HSPA1L* expression alone.

The interpretation of associations involving the *HSPA1L*/*HSPA1A* locus is further complicated by its location within the major histocompatibility complex (MHC) region, which is characterized by extensive linkage disequilibrium. Pairwise LD analysis revealed high D′ values but low-to-moderate r^2^ between the investigated variants in this region. Although the extensive eQTL associations observed for rs2227956 and rs2075799 may reflect underlying haplotype structure, LD with additional HLA and complement gene variants was not directly assessed. Therefore, the observed expression associations cannot be unequivocally attributed to independent regulatory effects of the investigated SNPs, and further fine-mapping studies are required to identify the underlying causal variants.

Protein–protein interaction analysis further identified several HSPA1L interaction partners implicated in processes relevant to IS pathogenesis, including DNAJB1, DNAJA1, DNAJA2, and TLR2 (Figure 3, Table S4). DNAJB1 and DNAJA1 participate in the biogenesis of apoB100, a major structural component of atherogenic lipoprotein particles, thereby providing a potential link between the HSPA1L interaction network and atherosclerosis [65]. Reduced DNAJA2 expression has also been reported in angiotensin II-induced endothelial dysfunction [64]. TLR2, in turn, contributes to inflammatory responses and apoptosis in ischemic tissues and may therefore link HSPA1L-associated protein networks to mechanisms of ischemic injury [66]. Collectively, the eQTL and protein-interaction data suggest that genetic variation at the *HSPA1L* locus may be connected to multiple processes relevant to IS, including atherogenesis, endothelial dysfunction, inflammation, and mitochondrial quality control.

Our study also revealed associations between *HSPA4* variants and clinical characteristics of IS. The rs13161158 TT genotype was associated with higher PTI values among individuals with low physical activity and low fruit and vegetable intake, whereas rs4616886 was associated with age at IS onset, aPTT, and BMI in subgroups stratified by lifestyle-related risk factors. These associations may be biologically relevant given the role of HSPA4 in cellular stress responses and autophagy. In *Hspa4*-knockout mice, accumulation of the autophagy markers LC3-II and p62 has been observed, consistent with impaired autophagic flux [67]. HSPA4 overexpression has also been reported to exert neuroprotective effects by suppressing ferroptosis and promoting neuronal survival [68].

The eQTL effects associated with rs4616886 *HSPA4* and rs13161158 *HSPA4* provide additional potential links to mitochondrial metabolism and cellular stress responses. Among their eQTL-associated genes, *SLC22A5* encodes a carnitine transporter required for mitochondrial fatty acid oxidation and maintenance of cellular energy homeostasis [69]. Another identified gene, *SLC22A4*, has previously been associated with reduced IS risk [70]. Its encoded transporter, OCTN1, mediates cellular uptake of ergothioneine, an antioxidant with potential protective effects against oxidative stress and inflammation, two processes central to both atherosclerosis and ischemic brain injury [71].

The HSPA4 protein-interaction network also contained several proteins implicated in mechanisms relevant to cerebral ischemia, including DNAJB1, STUB1, BAG3, HSPA8, and BAG1 (Figure 3, Table S4). Increased STUB1 expression has been reported to attenuate ischemia-induced neuronal degeneration and promote neuronal survival [72]. STUB1 also participates in the regulation of PINK1 through the STUB1–VCP/p97 complex, thereby linking this interaction network to mitophagy and mitochondrial quality control [73]. BAG3 has similarly been implicated in neuroprotection and in the regulation of autophagy, apoptosis, and inflammatory responses during ischemic injury [34,74,75,76]. HSPA8 has been associated with vascular inflammation [77,78] and atherosclerosis [79,80], as well as with cellular responses to oxidative stress in ischemic brain tissue [81]. BAG1 also regulates pathways involved in programmed cell death and glutamate excitotoxicity and has been reported to reduce cerebral infarct volume [35,82]. Together, these findings suggest that the functional network surrounding HSPA4 converges on several processes central to ischemic injury, particularly autophagy, mitochondrial quality control, oxidative stress, and neuronal survival.

The *HSPA6* variant rs753856 was associated with a reduced risk of IS in women and in subgroups defined by physical activity and fruit and vegetable intake. HSPA6 itself may contribute to vascular protection, as it has been shown to suppress arterial smooth muscle cell proliferation, a process involved in atherosclerotic vascular remodeling [27]. eQTL analysis further showed that rs753856 was associated with *FCGR2A* expression. *FCGR2A* has previously been identified as a gene shared between IS and myocardial ischemia, with elevated expression reported in the blood of patients with IS [83].

STRING analysis additionally identified DNAJA1, DNAJB1, TLR2, and MAPK9 among the functional interaction partners of HSPA6 (Figure 3, Table S4). While DNAJA1, DNAJB1, and TLR2 are discussed above, MAPK9 provides an additional potential link to neuronal apoptosis. MAPK9 can promote apoptotic signaling through phosphorylation of proteins including TP53 and YAP1. Because apoptosis contributes substantially to delayed neuronal loss in the ischemic penumbra, MAPK9-dependent signaling may participate in the progression of ischemic brain injury [84,85]. Thus, both the eQTL and protein-interaction findings support potential links between the *HSPA6* locus and pathways involved in vascular remodeling, inflammation, oxidative stress, and neuronal survival. However, the identified networks represent protein-level functional associations and do not provide direct evidence that the investigated genetic variants alter protein interactions. Therefore, these results should be considered complementary biological context.

BAG1 has been implicated in the regulation of apoptosis and excitotoxicity and has also been reported to reduce cerebral infarct volume [35,82]. In our study, the *BAG1* variant rs706121 was associated with IS risk in men. Protein–protein interaction analysis additionally identified several BAG1 partners with established roles in pathways relevant to cerebral ischemia, including DNAJB1, STUB1, BAG3, HSPA8, BAG2, and BCL2 (Figure 3, Table S4). The potential roles of DNAJB1, STUB1, BAG3, and HSPA8 are discussed above, whereas BAG2 and BCL2 provide additional links to mitochondrial homeostasis and neuronal survival. BAG2 has anti-apoptotic functions and can protect cells against oxidative stress while supporting mitochondrial quality control through stabilization of PINK1 and regulation of mitophagy [86,87]. Maintenance of efficient mitophagy is particularly important in neurons because removal of dysfunctional mitochondria is required to preserve mitochondrial function and ATP production [88]. BCL2 similarly promotes neuronal survival, and its overexpression has been shown to reduce ischemic brain injury [89]. In addition to its central role in apoptosis, BCL2 has been implicated in excitotoxicity, oxidative stress, mitochondrial dysfunction [90], inflammation, and atherosclerosis [91]. Together, these interactions support a potential connection between the BAG1 network and mechanisms regulating neuronal survival and mitochondrial integrity during cerebral ischemia.

The *BAG2* variant rs7749960 showed a protective effect against IS in the overall cohort and showed similar protective associations in men, smokers, and individuals with low physical activity or low fruit and vegetable intake. Given the anti-apoptotic and mitochondrial quality-control functions of BAG2 described above, these associations may be relevant to cellular resistance to ischemic stress. STRING analysis further identified BAG1, BAG3, HSPA8, and STUB1 among the major BAG2 interaction partners (Figure 3, Table S4). As these proteins converge on pathways regulating proteostasis, autophagy, apoptosis, and mitochondrial homeostasis, the BAG2 interaction network may contribute to mechanisms that determine cellular survival under ischemic conditions.

*BAG3* is another co-chaperone with reported neuroprotective functions in cerebral ischemia. Increased BAG3 expression following ischemic brain injury promotes autophagy and the clearance of damaged or aggregated proteins and has been associated with reduced apoptosis and smaller infarct volumes [74,75,76]. BAG3 may also modulate post-ischemic inflammation: increased BAG3 expression has been shown to suppress NLRP3 inflammasome and caspase-1 activation and reduce secretion of the pro-inflammatory cytokines IL-1β and TNF-α [34]. Beyond cerebral ischemia, BAG3 contributes to vascular homeostasis by protecting endothelial cells against oxidative stress; BAG3 deficiency has been associated with increased vascular injury, whereas BAG3 overexpression attenuates these effects [92].

Of note, rs2234962 is a missense variant in *BAG3* that has previously been associated with dilated cardiomyopathy [93] and cardiac function [94]. In particular, the C allele has been associated with a reduced risk of idiopathic dilated cardiomyopathy, while associations with several measures of left ventricular function have also been reported [95]. BAG3 is involved in multiple processes essential for cardiomyocyte function, including sarcomeric stabilization, autophagy, and protection against apoptosis [96,97,98]. However, these established functions of BAG3 do not necessarily reflect the specific molecular consequences of rs2234962. Although cardiac dysfunction may contribute to thromboembolic stroke, rs2234962 was not associated with overall IS susceptibility in our cohort, and information on etiological stroke subtypes was unavailable. Therefore, a potential relationship between this variant, cardiac dysfunction, and cardioembolic IS remains hypothetical and requires further investigation.

In our study, rs196329 in *BAG3* showed a risk effect for IS risk in individuals with normal fruit and vegetable intake. The risk-associated A allele was also associated with higher triglyceride and total cholesterol levels. These findings suggest a potential link between the genetic association at the *BAG3* locus and lipid-related mechanisms contributing to vascular disease. Consistent with this possibility, rs196329 showed an eQTL association with *CYP51A1*, which encodes an enzyme involved in cholesterol biosynthesis. The observed eQTL association with *CYP51A1* provides a potential link to lipid-related pathways [99]; however, its functional relevance to the clinical associations identified in this study remains to be established.

The BAG3 interaction network additionally included BAG1, BAG2, STUB1, HSPA8, BCL2, and HSPB8 (Figure 4, Table S7), further highlighting convergence on proteostasis, apoptosis, and mitochondrial quality-control pathways. HSPB8 is of particular interest because increased HSPB8 expression has been reported to attenuate mitochondria-dependent apoptotic signaling and promote neuronal survival [100]. In addition, Kobzeva et al. reported an association between the *HSPB8* variant rs1133026 and increased IS risk and later disease onset [101]. Taken together, the genetic, eQTL, and protein-interaction findings suggest that BAG3-related pathways may contribute to IS through a combination of lipid metabolism, inflammatory regulation, proteostasis, and neuronal stress resistance.

The targeted genotype–expression analysis identified a genotype-by-time association between rs1042665 and *HSPA9* expression: each additional rs1042665 C allele was associated with a greater IS-to-ISR expression change. *HSPA9*, also known as mortalin, is a predominantly mitochondrial HSP70-family member involved in mitochondrial protein import and folding, maintenance of mitochondrial proteostasis, and cellular responses to metabolic and oxidative stress [102,103]. This finding is compatible with genotype-dependent regulation of the *HSPA9* response during the first week after stroke; however, it does not establish the underlying mechanism and should be considered hypothesis-generating.

The rs1043618 C allele was associated with higher C4B expression during acute IS and lower *DDR1* expression on day 7. *C4B* encodes a component of the complement system [104], providing a potential link to acute innate immune and inflammatory responses. *DDR1* has been implicated in blood–brain barrier disruption, extracellular-matrix remodeling, and inflammatory responses after cerebral ischemia [48,105], as well as in atherogenesis, as discussed above. Importantly, significance at one time point but not the other does not itself demonstrate a difference between time points, and the formal genotype-by-time tests for *C4B* and *DDR1* did not remain significant after Benjamini–Hochberg correction. Furthermore, genotype-associated expression differences observed at individual time points do not necessarily indicate genotype-dependent changes during stroke recovery. These time-point-specific associations should therefore not be interpreted as evidence of phase-dependent genetic effects and require validation in larger longitudinal cohorts.

## 4. Materials and Methods

### 4.1. Study Population

The study included 1562 unrelated residents of Central Russia, comprising 768 patients with ischemic stroke (IS) and 794 control participants. The study was approved by the Ethics Committee of Kursk State Medical University, and all participants provided written informed consent.

The general clinical characteristics of the IS and control groups are presented in Table S10.

Biological samples were collected during two recruitment periods at two medical facilities in Kursk: from 2010 to 2012 at the Neurology Department of Kursk Emergency Hospital [106,107] and from 2015 to 2017 at the Regional Vascular Center of Kursk Regional Multidisciplinary Hospital [108,109]. The principal inclusion criterion was a confirmed diagnosis of IS established by a neurologist during the acute phase of the disease based on clinical and neurological examination and verified by computed tomography (CT) or magnetic resonance imaging (MRI) of the brain performed within 48 h of the onset of neurological symptoms. Patients with hemorrhagic stroke, secondary intracerebral hemorrhage, or a history of traumatic brain injury were excluded. All patients included in the study had documented arterial hypertension and were receiving antihypertensive therapy [110].

The control group consisted of healthy volunteers recruited from the same population and during the same period as the IS patients [111,112,113]. Controls were recruited at healthcare facilities in the Kursk region during routine medical examinations at state institutions. Individuals were included in the control group if they were not receiving antihypertensive therapy, had no clinical evidence of cardiovascular, cerebrovascular, or other severe diseases, and had normal blood pressure. Normal blood pressure was defined as systolic blood pressure < 130 mmHg and diastolic blood pressure < 85 mmHg, based on at least three separate measurements.

Data on physical activity and fruit and vegetable consumption were obtained from patients with ischemic stroke during their hospitalization. The aim was to analyze the level of physical activity and level of fruit and vegetable intake prior to the cerebrovascular event. Insufficient physical activity was defined as less than 180 min per week of moderate-to-vigorous physical activity, including leisure-time activities (e.g., walking and running) and structured exercise (e.g., treadmill running, aerobics, and resistance training). In accordance with World Health Organization (WHO) recommendations, low fruit and vegetable consumption was defined as an intake of less than 400 g per day, excluding potatoes and other starchy tubers. Corresponding lifestyle information was unavailable for the control group.

### 4.2. Selection of Candidate Genes and Single-Nucleotide Polymorphisms

The analysis included the *HSPA1A*, *HSPA1L*, *HSPA2*, *HSPA4*, *HSPA5*, *HSPA6*, *HSPA9*, and *HSPA14* genes, encoding members of the HSP70 family, as well as *BAG1*, *BAG2*, *BAG3*, *BAG4*, and *BAG5*, encoding HSP70-associated co-chaperones.

The study focused on a selected subset of *HSP70* family genes and BAG co-chaperones with potential relevance to cellular stress responses and ischemic injury. The investigated SNPs were prioritized based on a minor allele frequency of at least 0.05 in populations of European ancestry and evidence of regulatory potential (Table S11). The study was designed as a targeted candidate-variant analysis rather than a comprehensive assessment of genetic variation across these loci. Accordingly, the investigated variants provide only partial coverage of the selected genes, and the findings cannot exclude associations involving other variants or unexamined HSP70 family members.

### 4.3. DNA Extraction and Genotyping

Genotyping was performed at the Laboratory of Genomic Research, Research Institute of Genetic and Molecular Epidemiology, Kursk State Medical University (Kursk, Russia). Genomic DNA was extracted from thawed blood samples, and DNA quality was assessed using a NanoDrop spectrophotometer (Thermo Fisher Scientific, Waltham, MA, USA).

SNPs rs753856 *HSPA6*, rs706121 *BAG1*, rs196336 and rs196329 *BAG3*, rs11625397 *BAG5*, rs1043618 *HSPA1A*, rs13161158 *HSPA4* and rs1042665 *HSPA9*, and rs17155992 *HSPA14* were genotyped by PCR using TaqMan probe technology, described in detail previously [114]. SNPs rs3778515 and rs7749960 *BAG2*, rs196345, rs7099687, rs196325, and rs2234962 *BAG3*, rs12682626 *BAG4*, rs2227956 and rs2075799 *HSPA1L*, rs1063391 *HSPA2*, rs12173082, rs4616886, and rs11749966 *HSPA4*, and rs12009 and rs430397 *HSPA5* were genotyped using iPLEX technology on the MassARRAY 4 system (Agena Bioscience, San Diego, CA, USA) [115]. The structure of the primers used for genotyping on the MassARRAY 4 platform is presented in Table S12.

For genotyping quality control, 10% of DNA samples were analyzed in duplicate in a blinded manner, without information on case–control status. The concordance rate between replicate genotypes exceeded 99%. Genotype frequencies for all studied SNPs and their distribution’s correspondence to the Hardy–Weinberg equilibrium are presented in Table S13. SNP rs7099687 *BAG3* was excluded from subsequent analyses because its genotype distribution deviated from Hardy–Weinberg equilibrium in controls.

### 4.4. Statistical Analysis

Statistical power was assessed using the Genetic Association Study (GAS) Power Calculator (https://csg.sph.umich.edu/abecasis/gas_power_calculator/, accessed 4 June 2026) [116]. Based on a sample of 768 patients with IS and 794 controls, the minimum detectable genotype relative risk was estimated across the range of allele frequencies investigated in this study. Assuming 80% statistical power, a significance level of α = 0.05, the estimated minimum detectable genotype relative risk ranged from 1.22 to 1.51.

Associations between SNPs and IS risk were evaluated under a log-additive genetic model using PLINK (v1.9.0-b.7.7, 22 October 2024; https://www.cog-genomics.org/plink/1.9/). The analyses were adjusted for covariates (sex, age and smoking) accounting for differences in the general biological and clinical characteristics of the compared groups.

Multiple-testing correction was performed using an adaptive permutation procedure, and permutation-adjusted *p*-values (*p_perm_* < 0.05) were considered statistically significant. When information on a particular clinical or environmental characteristic was unavailable for the control group, IS patient subgroups stratified according to the corresponding risk factor were compared with the entire control group, with Bonferroni correction applied for multiple comparisons. These analyses were considered subgroup-specific case–control comparisons and were not interpreted as formal tests of gene–environment interaction, for which exposure information in both cases and controls would be required.

Pairwise linkage disequilibrium (LD) between the investigated SNPs located on the same chromosome was assessed using PLINK v1.9 with the ‘--r2 dprime’ option. LD was quantified using the squared correlation coefficient (r^2^) and the standardized disequilibrium coefficient (D′).

To assess potential differences in genetic associations by sex and smoking status, subgroup-specific logistic regression models were fitted for each SNP, with adjustment for age and the remaining covariate (smoking status or sex, respectively). Heterogeneity between subgroup-specific log-odds ratios was evaluated using Cochran’s Q test. The resulting heterogeneity *p*-values were adjusted for multiple testing across SNPs using the Benjamini–Hochberg false-discovery rate (FDR) procedure separately for sex and smoking status.

Comparisons of quantitative variables between groups were performed in R version 4.4.1, R Foundation for Statistical Computing, Vienna, Austria). Differences among three groups were assessed using the Kruskal–Wallis test, followed by pairwise Mann–Whitney U tests. Data were visualized using the *ggplot2* (version 4.0.2) and *ggpubr* (version 0.6.3) R packages.

### 4.5. Functional Annotation and eQTL Analysis

The potential functional consequences of the investigated SNPs were assessed using a combination of bioinformatic approaches described previously [117,118].

Publicly available expression quantitative trait locus (eQTL) data were used to investigate associations between the studied variants and gene expression. The GTEx portal (version 11) was queried for eQTL associations in whole blood, vascular tissues, and brain tissues (GTEx Consortium 2020), while the eQTLGen Consortium browser/database (Phase 2; https://www.eqtlgen.org/, accessed 10 September 2026) was used to assess eQTL effects in peripheral blood [119].

Protein-level functional associations were examined using STRING (version 12.5) [120] for HSPA1L, HSPA4, HSPA6, BAG1, BAG2, and BAG3, selected based on genetic associations with IS risk or clinical characteristics. The full STRING network was used with a minimum combined confidence score of 0.400 (medium confidence). All available evidence sources were enabled, including text mining, experiments, databases, co-expression, genomic neighborhood, gene fusion, co-occurrence, and evidence transfer. Network visualizations were restricted to a maximum of 10 first-shell interactors per query protein, with no second-shell interactors. Combined confidence scores were recorded for each association. *HSPA14* was included in the genetic screening but was not selected for network visualization because no association with IS was identified. The resulting networks were used to provide functional context rather than to assess variant-specific effects on protein interactions.

### 4.6. Targeted Genotype–Expression Analysis in the Ischemic Stroke RNA-Seq Cohort

To complement the publicly available eQTL data, a targeted genotype–expression analysis was performed using paired whole-blood RNA-seq samples from 19 patients with IS. Whole-blood samples were collected from 19 patients with CT-confirmed IS at the Regional Vascular Center of Kursk Regional Hospital (Kursk, Russia) in 2025–2026. Samples were obtained at two time points: within the first 24 h of hospitalization (acute phase) and on day 7 of hospitalization (early post-stroke phase), yielding 38 paired RNA-seq samples. Patients with intracerebral hemorrhage, hemodynamic- or dissection-related stroke, traumatic brain injury, hepatic or renal failure, autoimmune or oncological disease, or other conditions associated with acute cerebrovascular events were excluded.

Total RNA was extracted from whole blood using the R-Blood-50 kit (Biolabmix, Novosibirsk, Russia) according to the manufacturer’s instructions. RNA concentration was measured using a Qubit 4 fluorometer (Thermo Fisher Scientific, USA), and RNA integrity was assessed using RNA integrity numbers (RIN). Libraries were prepared from 250 ng of total RNA using poly(A) enrichment without globin-RNA depletion and the VAHTS Universal V8 RNA-seq Library Prep Kit for Illumina (Vazyme, Nanjing, China). Paired-end sequencing (2 × 51 bp) was performed on an Illumina NextSeq 2000 platform (Illumina, San Diego, CA, USA).

RNA-seq data were processed using the Illumina DRAGEN Bio-IT Platform (version 3.9.5), with gene-level expression quantification performed using the DRAGEN RNA pipeline. Sequencing and alignment quality were evaluated using Qualimap (version 2.3) and MultiQC (version 1.14), including assessment of read mapping, duplication rates, insert lengths, GC content, and base quality.

In the present study, these transcriptomic profiles were used for targeted genotype–expression analyses of selected HSP70-related variants, as described below.

Genotypes were represented as additive dosages of the alternative allele, corresponding to 0, 1, or 2 copies. Three variants for which both genotype and transcriptomic data were available were investigated: rs753856 *HSPA6*, rs1042665 *HSPA9*, and rs1043618 *HSPA1A*.

Candidate SNP–gene pairs were restricted to expressed genes whose transcription start sites were located within 1 Mb of the corresponding SNP. Nine SNP–gene pairs were prioritized based on three criteria: (i) the corresponding HSP70 family genes (*HSPA6*, *HSPA9*, and *HSPA1A*); (ii) four genes (*FCGR2A*, *FCGR2B*, *FCGR2C*, and *FCRLB*) supported by previously reported eQTL associations with rs753856 in the eQTLGen database; and (iii) two additional genes (*C4B* and *DDR1*) prioritized for rs1043618 based on biological relevance and independent GTEx/brain-related evidence, respectively. The complete list of selected SNP–gene pairs, their selection criteria, and distances from the corresponding SNPs are provided in Table S14.

For normalization and dispersion estimation in DESeq2 (version 1.46.0), the expression dataset was filtered to retain genes with a raw read count ≥10 in at least 10 of the 38 samples. Statistical testing was subsequently restricted to the nine predefined SNP–gene pairs.

Three hypotheses were evaluated: (i) whether gene expression during the acute phase of IS was associated with genotype dosage; (ii) whether gene expression on day 7 was associated with genotype dosage; and (iii) whether genotype dosage was associated with the magnitude of gene-expression change between the acute phase and day 7.

For the cross-sectional analyses at each individual time point, the following model was fitted:

*expression ~ genotype_dosage*

For the longitudinal analysis, the paired structure of the dataset was accounted for using the following model:

*expression ~ patient + timepoint + genotype_time*

where ‘patient’ represents the individual patient identifier and ‘timepoint’ distinguishes the acute IS and day-7 ISR samples. The ‘genotype_time’ variable was defined as 0 for the acute IS sample and as the corresponding genotype dosage for the day-7 ISR sample. Thus, the coefficient for ‘genotype_time’ estimates the genotype-dependent difference in expression change between the two time points.

For each of the three hypotheses, *p*-values obtained for the nine predefined SNP–gene pairs were corrected separately for multiple testing using the Benjamini–Hochberg false-discovery rate procedure.

## 5. Conclusions

In conclusion, this study provides a comprehensive assessment of the contribution of genetic variation in HSP70 family genes and their co-chaperones to IS. Several investigated variants were associated with IS susceptibility (rs2227956 *HSPA1L*, rs7749960 *BAG2*) and with clinical and biochemical characteristics, including age at disease onset (rs4616886 and rs11749966 *HSPA4*, rs706121 *BAG1*), lipid levels (rs196329 *BAG3*, rs196329 *BAG3*), coagulation parameters (rs4616886 and rs13161158 *HSPA4*, rs2075799 *HSPA1L*), and BMI (rs2234962 *BAG3*, rs4616886 *HSPA4*). Additional associations observed in subgroups stratified by sex (rs2227956 and rs2075799 *HSPA1L*, rs753856 *HSPA6*, rs706121 *BAG1*, rs7749960 *BAG2*) and lifestyle-related risk factors (rs2227956 *HSPA1L*, rs753856 *HSPA6*, rs7749960 *BAG2*, rs196329 *BAG3*) suggest that the effects of HSP70-related genetic variation may depend on both biological and environmental context.

Functional analyses identified extensive eQTL associations and protein-interaction networks involving pathways relevant to IS pathogenesis, including atherosclerosis, inflammation, apoptosis, oxidative stress, autophagy, mitochondrial dysfunction, and angiogenesis. Moreover, targeted analysis of paired blood transcriptomic profiles indicated that selected variants were associated with gene-expression differences during the acute and subacute phases of IS, suggesting that their functional effects may vary over the course of ischemic injury.

Taken together, these findings support a potential role of HSP70-related genetic variation in IS susceptibility and clinical phenotype and provide a basis for further investigation of these loci as biomarkers of disease risk, outcome, and molecular response to ischemic injury. Validation in larger and independent cohorts will be required to establish their potential utility in risk stratification IS.

## 6. Study Limitations

Several limitations should be acknowledged. First, the selection of candidate genes and variants did not comprehensively cover genetic variation across the HSP70 family. Furthermore, the case and control groups differed substantially in their cardiovascular risk profiles. All patients with IS had treated arterial hypertension, whereas controls were normotensive and free of cardiovascular disease. Consequently, hypertension could not be included as an independent covariate, and some observed genetic associations may reflect susceptibility to hypertension or broader cardiovascular disease rather than IS specifically. Although the analyses were adjusted for age, sex, and smoking status, residual confounding cannot be excluded. Additionally, the younger age of the control group raises the possibility that some participants may develop IS later in life.

Other limitations of our study are the use of a sample from a single population and the absence of independent replication. Furthermore, for the SNPs analysis, we limited ourselves to an adaptive permutation test and did not apply an additional correction for multiple comparisons.

Information on physical activity and fruit and vegetable consumption was unavailable for controls, while data on IS subtypes and lipid-lowering therapy were unavailable for patients. The absence of etiological stroke classification (e.g., TOAST) prevented subtype-specific analyses; therefore, the proposed relationships between the investigated variants and atherosclerotic or cardioembolic mechanisms remain speculative. These limitations restricted covariate adjustment and the assessment of gene–environment interactions.

Finally, the targeted genotype–expression analysis included only 19 patients, with particularly small genotype groups for rs753856, limiting statistical power and the precision of effect estimates. Despite correction for multiple testing, chance findings cannot be excluded. Moreover, peripheral-blood expression may not fully reflect regulatory processes in brain tissue, and the observational analysis does not establish causality. Therefore, the findings require validation in larger independent cohorts, including hypertensive controls without IS, and further functional studies.

## Figures and Tables

**Table 1:** Results of the analysis of associations between *HSP70* SNPs and risk of IS in the entire group.

Genetic Variant	Effect Allele (Frequency)	N	OR [95% CI] ^1^	*p_perm_* ^2^
rs1043618 *HSPA1A* (G/C)	C (0.35 ^IS^/0.36 ^C^)	1544	0.96 [0.82–1.13]	0.73
rs2227956 *HSPA1L* (G/A)	G (0.15 ^IS^/0.19 ^C^)	1315	**0.66 [0.53–0.83]**	**0.0004**
rs2075799 *HSPA1L* (C/T)	T (0.13 ^IS^/0.11 ^C^)	1538	1.19 [0.94–1.51]	0.16
rs1063391 *HSPA2* (C/T)	C (0.32 ^IS^/0.33 ^C^)	1527	1.01 [0.85–1.19]	1.00
rs12173082 *HSPA4* (C/G)	G (0.22 ^IS^/0.23 ^C^)	1541	0.96 [0.80–1.14]	0.86
rs13161158 *HSPA4* (T/C)	C (0.06 ^IS^/0.07 ^C^)	1558	1.14 [0.84–1.54]	0.36
rs4616886 *HSPA4* (T/C)	C (0.14 ^IS^/0.14 ^C^)	1461	0.92 [0.74–1.15]	0.59
rs11749966 *HSPA4* (T/C)	C (0.05 ^IS^/0.04 ^C^)	1552	1.40 [0.98–2.00]	0.05
rs12009 *HSPA5* (G/A)	G (0.44 ^IS^/0.42 ^C^)	1555	1.07 [0.92–1.25]	0.35
rs430397 *HSPA5* (C/T)	T (0.08 ^IS^/0.09 ^C^)	1545	0.98 [0.75–1.28]	1.00
rs753856 *HSPA6* (C/G)	G (0.09 ^IS^/0.12 ^C^)	1548	0.79 [0.61–1.02]	0.12
rs1042665 *HSPA9* (T/C)	C (0.22 ^IS^/0.22 ^C^)	1543	1.04 [0.86–1.26]	1.00
rs17155992 *HSPA14* (G/A)	A (0.08 ^IS^/0.07 ^C^)	1550	1.07 [0.81–1.42]	0.63
rs706121 *BAG1* (C/T)	C (0.16 ^IS^/0.15 ^C^)	1518	1.01 [0.81–1.24]	0.86
rs3778515 *BAG2* (T/G)	G (0.24 ^IS^/0.24 ^C^)	1443	1.05 [0.87–1.26]	0.52
rs7749960 *BAG2* (C/A)	A (0.09 ^IS^/0.13 ^C^)	1545	**0.72 [0.56–0.92]**	**0.01**
rs196336 *BAG3* (T/C)	T (0.42 ^IS^/0.42 ^C^)	1550	0.98 [0.84–1.15]	0.86
rs196345 *BAG3* (G/A)	A (0.36 ^IS^/0.35 ^C^)	1429	1.11 [0.94–1.30]	0.19
rs196325 *BAG3* (A/G)	A (0.42 ^IS^/0.42 ^C^)	1554	1.02 [0.87–1.19]	0.86
rs2234962 *BAG3* (T/C)	C (0.21 ^IS^/0.20 ^C^)	1548	1.09 [0.90–1.31]	0.86
rs196329 *BAG3* (A/G)	A (0.26 ^IS^/0.24 ^C^)	1539	1.12 [0.94–1.33]	0.26
rs12682626 *BAG4* (A/G)	G (0.22 ^IS^/0.22 ^C^)	1550	0.99 [0.82–1.19]	1.00
rs11625397 *BAG5* (C/T)	C (0.33 ^IS^/0.32 ^C^)	1451	1.04 [0.87–1.23]	0.78

All calculations were performed relative to the minor alleles (Effect allele) with adjustment for sex, age and smoking; 1—odds ratio and 95% confidence interval; 2—*p*-value after permutation test. IS—patients; C—controls; statistically significant associations are highlighted in bold.

**Table 2:** Statistically significant associations between *HSP70* SNPs and IS risk in sex-specific subgroups.

Genetic Variant	Effect Allele (Frequency)	N	OR [95% CI] ^1^	*p_perm_* ^2^	Effect Allele (Frequency)	N	OR [95% CI] ^1^	*p_perm_* ^2^
	Males (n = 724: 428 ^IS^; 296 ^C^)				Females (n = 838: 340 ^IS^; 498 ^C^)			
rs2227956 *HSPA1L* (G/A)	G (0.16 ^IS^/0.22 ^C^)	597	**0.63** **[0.47–0.86]**	**0.003**	G (0.14 ^IS^/0.17 ^C^)	718	0.75 [0.56–1.02]	0.08
rs2075799 *HSPA1L* (C/T)	T (0.13 ^IS^/0.09 ^C^)	713	**1.53** **[1.08–2.17**]	**0.02**	T (0.13 ^IS^/0.12 ^C^)	825	1.04 [0.77–1.39]	0.73
rs753856 *HSPA6* (C/G)	G (0.10 ^IS^/0.12 ^C^)	719	0.82 [0.58–1.16]	0.28	G (0.09 ^IS^/0.12 ^C^)	829	**0.70** **[0.50–0.98]**	**0.03**
rs706121 *BAG1* (C/T)	C (0.18 ^IS^/0.13 ^C^)	705	**1.40** **[1.04–1.89]**	**0.02**	C (0.13 ^IS^/0.16 ^C^)	813	0.79 [0.59–1.04]	0.08
rs7749960 *BAG2* (C/A)	A (0.08 ^IS^/0.13 ^C^)	716	**0.58** **[0.41–0.82]**	**0.002**	A (0.11 ^IS^/0.13 ^C^)	829	0.82 [0.60–1.11]	0.23

All calculations were performed relative to the minor alleles (Effect allele) with adjustment for age and smoking; 1—odds ratio and 95% confidence interval; 2—*p*-value after permutation test. IS—patients; C—controls; statistically significant associations are highlighted in bold.

**Table 3:** Results of the analysis of associations between *HSP70* SNPs and IS risk in groups stratified by smoking status.

Genetic Variant	Effect Allele (Frequency)	N	OR [95% CI] ^1^	*p_perm_* ^2^	Effect Allele (Frequency)	N	OR [95% CI] ^1^	*p_perm_* ^2^
	Smokers (n = 584: 387 ^IS^; 197 ^C^)				Nonsmokers (n = 978: 381 ^IS^; 597 ^C^)			
rs2227956 (G/A) *HSPA1L*	G (0.17 ^IS^/0.17 ^C^)	478	0.97 [0.67–1.41]	0.86	G (0.13 ^IS^/0.20 ^C^)	837	**0.60** **[0.45–0.79]**	**0.0002**
rs7749960 (C/A) *BAG2*	A (0.08 ^IS^/0.15 ^C^)	576	**0.50** **[0.34–0.74]**	**0.001**	A (0.11 ^IS^/0.13 ^C^)	969	0.83 [0.62–1.10]	0.19

All calculations were performed relative to the minor alleles (Effect allele) with adjustment for sex, age; 1—odds ratio and 95% confidence interval; 2—*p*-value after permutation test; IS—patients; C—controls; statistically significant associations are highlighted in bold.

**Table 4:** Results of the analysis of associations between *HSP70* SNPs and IS risk in groups stratified by physical activity, fruit/vegetable intake.

Genetic Variant	Effect Allele (Frequency)	N	OR [95% CI] ^1^	*p_perm_*^2^ (*^p^_bonf_*)	Effect Allele (Frequency)	N	OR [95% CI] ^1^	*p_perm_*^2^ (*^p^_bonf_*)
	Low physical activity level (n = 1084: 290 ^IS^; 794 ^C^)				Normal physical activity level (n = 974: 180 ^IS^; 794 ^C^)			
rs2227956 (G/A) *HSPA1L*	G (0.13 ^IS^/0.19 ^C^)	911	**0.66** **[0.49–0.88]**	**0.005 (0.01)**	G (0.15 ^IS^/0.19 ^C^)	818	0.78 [0.55–1.10]	0.17 (0.34)
rs753856 (C/G) *HSPA6*	G (0.10 ^IS^/0.12 ^C^)	1073	0.85 [0.62–1.16]	0.38 (0.76)	G (0.08 ^IS^/0.12 ^C^)	965	**0.60** **[0.40–0.91]**	0.02 (0.04)
rs7749960 (C/A) *BAG2*	A (0.09 ^IS^/0.13 ^C^)	1074	**0.69** **[0.50–0.95]**	**0.02 (0.04)**	A (0.11 ^IS^/0.13 ^C^)	966	0.83 [0.58–1.19]	0.26 (0.52)
	Low fruit/vegetable intake (n = 1187: 393 ^IS^; 794 ^C^)				Normal fruit/vegetable intake (n = 1169: 375 ^IS^; 794 ^C^)			
rs2227956 (G/A) *HSPA1L*	G (0.16 ^IS^/0.19 ^C^)	998	0.78 [0.60–1.00]	0.04 (0.08)	G (0.14 ^IS^/0.19 ^C^)	982	**0.69** **[0.53–0.90]**	**0.005 (0.01)**
rs753856 (C/G) *HSPA6*	G (0.09 ^IS^/0.12 ^C^)	1176	**0.69** **[0.51–0.93]**	**0.02 (0.04)**	G (0.10 ^IS^/0.12 ^C^)	1158	0.85 [0.64–1.13]	0.36 (0.72)
rs7749960 (C/A) *BAG2*	A (0.09 ^IS^/0.13 ^C^)	1176	**0.62** **[0.47–0.84]**	**0.001 (0.002)**	A (0.10 ^IS^/0.13 ^C^)	1157	0.75 [0.57–0.99]	0.04 (0.08)
rs196329 (A/G) *BAG3*	A (0.24 ^IS^/0.24 ^C^)	1179	1.01 [0.82–1.23]	1.00 (2.00)	A (0.28 ^IS^/0.24 ^C^)	1154	**1.25** **[1.03–1.52]**	**0.02 (0.04)**

All calculations were performed relative to the minor alleles (Effect allele) with adjustment for sex, age, smoking; 1—odds ratio and 95% confidence interval; 2—*p*-value after permutation test; bonf (Bonferroni correction); IS—patients; C—controls (overall group); statistically significant associations are highlighted in bold.

## Data Availability

Summary statistics supporting the genetic association analyses, including genotype distributions in patients with IS and controls, are provided in the Supplementary Materials. Due to current local legal and institutional restrictions governing the sharing of human genomic data, the raw RNA-seq datasets cannot be deposited in a public repository at this time. Requests for additional data may be directed to the corresponding author and will be considered in accordance with applicable legal requirements, institutional regulations, and participant consent.

## Supplementary Materials

The following supporting information can be downloaded at https://www.mdpi.com/article/10.3390/ijms27219560/s1.
