# Supplementary material for: Genetic Variants in HSP70 Family Genes and Their Co-Chaperones Are Associated with Ischemic Stroke Susceptibility and Gene Expression During Acute and Early Recovery Phases
Source: Int J Mol Sci. 2026 Oct 26;27(21):9560. doi: 10.3390/ijms27219560 (PMC13762509; doi:10.3390/ijms27219560)
Supplement: Supplementary file 1 [file ijms-27-09560-s001.zip › ijms-4609126-supplementary 11.10.2026.pdf]

Table S1. Pairwise linkage disequilibrium between investigated HSP70-related genetic variants located on the same chromosome

Chr	SNP_A	Gene_A	SNP_B	Gene_B	Distance (kb)	R^2	D'
5	rs11749966	*HSPA4*	rs1042665	*HSPA9*	5478.3	0	0.009
5	rs12173082	*HSPA4*	rs1042665	*HSPA9*	5512.7	0.002	0.158
5	rs12173082	*HSPA4*	rs11749966	*HSPA4*	34.4	0.015	1
5	rs12173082	*HSPA4*	rs13161158	*HSPA4*	18	0.022	1
5	rs12173082	*HSPA4*	rs4616886	*HSPA4*	34.1	0.05	1
5	rs13161158	*HSPA4*	rs1042665	*HSPA9*	5494.7	0	0.065
5	rs13161158	*HSPA4*	rs11749966	*HSPA4*	16.4	0.003	0.916
5	rs13161158	*HSPA4*	rs4616886	*HSPA4*	16.1	0.01	0.912
5	rs4616886	*HSPA4*	rs1042665	*HSPA9*	5478.6	0.001	0.142
5	rs4616886	*HSPA4*	rs11749966	*HSPA4*	0.3	0.008	1
6	rs1043618	*HSPA1A*	rs3778515	*BAG2*	25362.2	0.006	0.185
6	rs1043618	*HSPA1A*	rs7749960	*BAG2*	25365	0	0.044
6	rs2075799	*HSPA1L*	rs1043618	*HSPA1A*	5	0.214	0.943
6	rs2075799	*HSPA1L*	rs3778515	*BAG2*	25367.2	0.001	0.149
6	rs2075799	*HSPA1L*	rs7749960	*BAG2*	25370	0	0.022
6	rs2227956	*HSPA1L*	rs1043618	*HSPA1A*	5.2	0.095	0.919
6	rs2227956	*HSPA1L*	rs2075799	*HSPA1L*	0.3	0.028	1
6	rs2227956	*HSPA1L*	rs3778515	*BAG2*	25367.4	0.001	0.046
6	rs2227956	*HSPA1L*	rs7749960	*BAG2*	25370.3	0	0.012
6	rs3778515	*BAG2*	rs7749960	*BAG2*	2.8	0.423	0.996
9	rs12009	*HSPA5*	rs430397	*HSPA5*	3.8	0.127	1
9	rs706121	*BAG1*	rs12009	*HSPA5*	91974.4	0	0.021
9	rs706121	*BAG1*	rs430397	*HSPA5*	91978.2	0	0.006
10	rs17155992	*HSPA14*	rs196325	*BAG3*	104819.9	0.001	0.136
10	rs17155992	*HSPA14*	rs196329	*BAG3*	104823.6	0.002	0.257
10	rs17155992	*HSPA14*	rs196336	*BAG3*	104805.3	0.002	0.16
10	rs17155992	*HSPA14*	rs196345	*BAG3*	104812	0.001	0.173
10	rs17155992	*HSPA14*	rs2234962	*BAG3*	104820.7	0	0.082
10	rs196325	*BAG3*	rs196329	*BAG3*	3.7	0.077	0.567
10	rs196325	*BAG3*	rs2234962	*BAG3*	0.8	0.234	0.805
10	rs196336	*BAG3*	rs196325	*BAG3*	14.6	0.021	0.145
10	rs196336	*BAG3*	rs196329	*BAG3*	18.3	0.371	0.895
10	rs196336	*BAG3*	rs196345	*BAG3*	6.7	0.354	0.938
10	rs196336	*BAG3*	rs2234962	*BAG3*	15.4	0.175	0.969
10	rs196345	*BAG3*	rs196325	*BAG3*	7.9	0.061	0.283
10	rs196345	*BAG3*	rs196329	*BAG3*	11.6	0.115	0.791
10	rs196345	*BAG3*	rs2234962	*BAG3*	8.7	0.462	0.994
10	rs2234962	*BAG3*	rs196329	*BAG3*	2.9	0.053	0.781
14	rs1063391	*HSPA2*	rs11625397	*BAG5*	39019.7	0	0.011

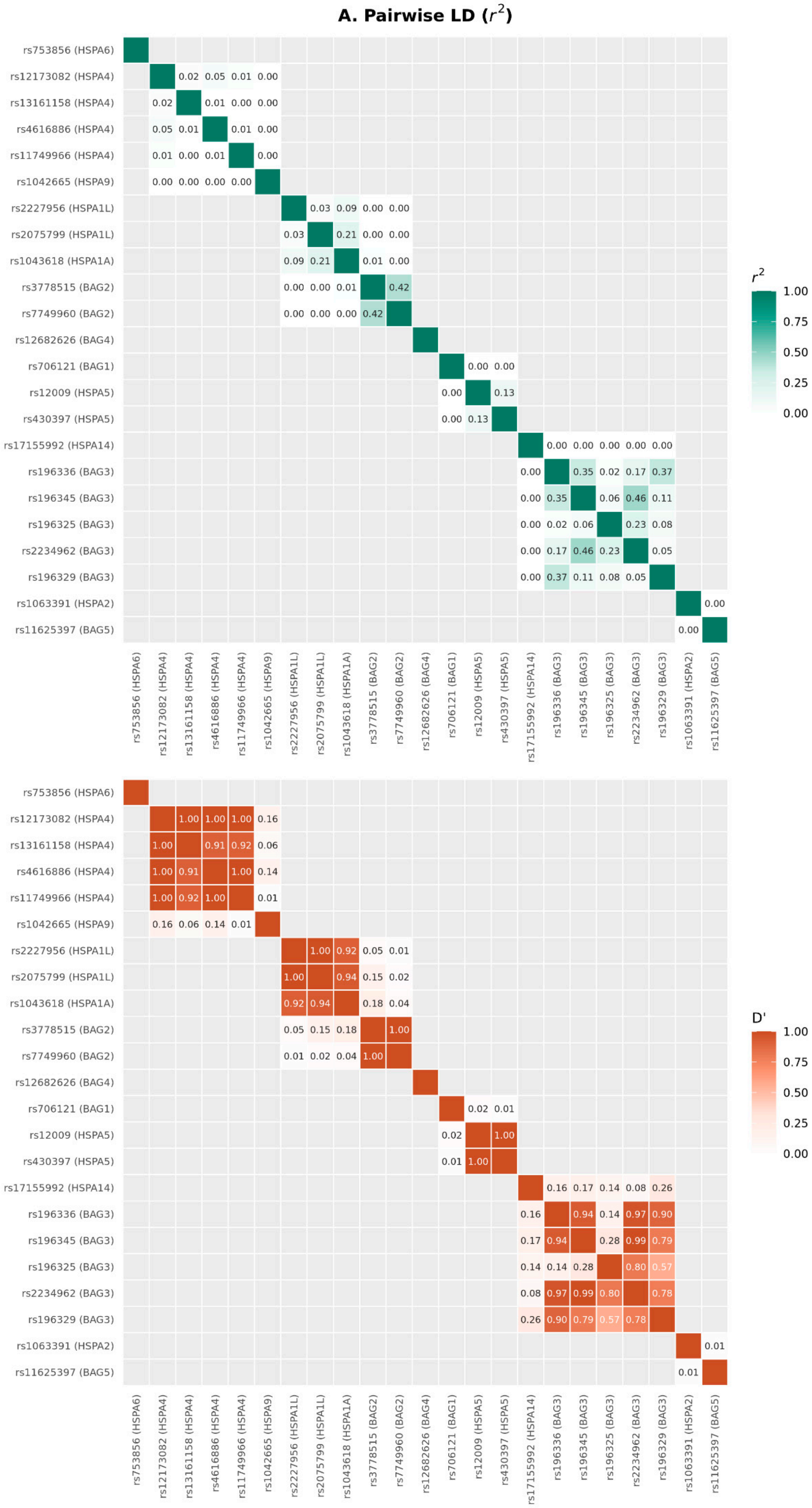
A. Pairwise LD (r^2)
rs753856 (HSPA6)
rs12173082 (HSPA4) 0.02 0.05 0.01 0.00
rs13161158 (HSPA4) 0.02 0.01 0.00 0.00
rs4616886 (HSPA4) 0.05 0.01 0.01 0.00
rs11749966 (HSPA4) 0.01 0.00 0.01 0.00
rs1042665 (HSPA9) 0.00 0.00 0.00 0.00
rs2227956 (HSPA1L) 0.03 0.09 0.00 0.00
rs2075799 (HSPA1L) 0.03 0.21 0.00 0.00
rs1043618 (HSPA1A) 0.09 0.21 0.01 0.00
rs3778515 (BAG2) 0.00 0.00 0.01 0.42
rs7749960 (BAG2) 0.00 0.00 0.00 0.42
rs12682626 (BAG4)
rs706121 (BAG1) 0.00 0.00
rs12009 (HSPA5) 0.00 0.13
rs430397 (HSPA5) 0.00 0.13
rs17155992 (HSPA14) 0.00 0.00 0.00 0.00 0.00
rs196336 (BAG3) 0.00 0.35 0.02 0.17 0.37
rs196345 (BAG3) 0.00 0.35 0.06 0.46 0.11
rs196325 (BAG3) 0.00 0.02 0.06 0.23 0.08
rs2234962 (BAG3) 0.00 0.17 0.46 0.23 0.05
rs196329 (BAG3) 0.00 0.37 0.11 0.08 0.05
rs1063391 (HSPA2) 0.00
rs11625397 (BAG5) 0.00
r^2
1.00
0.75
0.50
0.25
0.00
rs753856 (HSPA6)
rs12173082 (HSPA4) 1.00 1.00 1.00 0.16
rs13161158 (HSPA4) 1.00 0.91 0.92 0.06
rs4616886 (HSPA4) 1.00 0.91 1.00 0.14
rs11749966 (HSPA4) 1.00 0.92 1.00 0.01
rs1042665 (HSPA9) 0.16 0.06 0.14 0.01
rs2227956 (HSPA1L) 1.00 0.92 0.05 0.01
rs2075799 (HSPA1L) 1.00 0.94 0.15 0.02
rs1043618 (HSPA1A) 0.92 0.94 0.18 0.04
rs3778515 (BAG2) 0.05 0.15 0.18 1.00
rs7749960 (BAG2) 0.01 0.02 0.04 1.00
rs12682626 (BAG4)
rs706121 (BAG1) 0.02 0.01
rs12009 (HSPA5) 0.02 1.00
rs430397 (HSPA5) 0.01 1.00
rs17155992 (HSPA14) 0.16 0.17 0.14 0.08 0.26
rs196336 (BAG3) 0.16 0.94 0.14 0.97 0.90
rs196345 (BAG3) 0.17 0.94 0.28 0.99 0.79
rs196325 (BAG3) 0.14 0.14 0.28 0.80 0.57
rs2234962 (BAG3) 0.08 0.97 0.99 0.80 0.78
rs196329 (BAG3) 0.26 0.90 0.79 0.57 0.78
rs1063391 (HSPA2) 0.01
rs11625397 (BAG5) 0.01
D'
1.00
0.75
0.50
0.25
0.00

Figure S1. Pairwise linkage disequilibrium within the *HSPA1L*/*HSPA1A*, *HSPA4*, and *BAG3* genomic regions. Lower triangle displays r^2 and the upper triangle D′.

Table S2. Results of the analysis of associations between HSP70 SNPs and IS risk in groups stratified by sex.

Genetic variant	Effect allele (frequency)	N	OR [95% CI]1	p[2]	p_{perm}[3]	Effect allele (frequency)	N	OR [95% CI][1]	p[2]	p_{perm}[3]
	Males (n=724: 428^{IS}; 296^{C})					Females (n=838: 340^{IS}; 498^{C})				
rs1043618 *HSPA1A* (G/C)	C (0,33^{IS}/0,33^{C})	716	1.01 [0.81-1.26]	0.93	1.00	C (0,37^{IS}/0,38^{C})	828	0.93 [0.76-1.14]	0.51	0.41
rs2227956 *HSPA1L* (G/A)	G (0,16^{IS}/0,22^{C})	597	**0.63 [0.47-0.86]**	**0.003**	**0.003**	G (0,14^{IS}/0,17^{C})	718	0.75 [0.56-1.02]	0.06	0.08
rs2075799 *HSPA1L* (C/T)	T (0,13^{IS}/0,09^{C})	713	**1.53 [1.08-2.17]**	**0.02**	**0.02**	T (0,13^{IS}/0,12^{C})	825	1.04 [0.77-1.39]	0.82	0.73
rs1063391 *HSPA2* (C/T)	C (0,31^{IS}/0,31^{C})	706	1.02 [0.81-1.30]	0.85	0.86	C (0,33^{IS}/0,35^{C})	821	0.92 [0.75-1.13]	0.43	0.59
rs12173082 *HSPA4* (C/G)	G (0,22^{IS}/0,24^{C})	715	0.90 [0.71-1.15]	0.41	0.31	G (0,23^{IS}/0,23^{C})	826	0.96 [0.76-1.22]	0.75	0.86
rs13161158 *HSPA4* (T/C)	C (0,07^{IS}/0,06^{C})	722	1.21 [0.78-1.86]	0.39	0.41	C (0,08^{IS}/0,06^{C})	836	1.21 [0.83-1.78]	0.32	0.36
rs4616886 *HSPA4* (T/C)	C (0,14^{IS}/0,17^{C})	675	0.80 [0.60-1.06]	0.12	0.10	C (0,14^{IS}/0,12^{C})	786	1.26 [0.95-1.69]	0.11	0.10
rs11749966 *HSPA4* (T/C)	C (0,05^{IS}/0,05^{C})	720	1.09 [0.67-1.75]	0.74	0.63	C (0,05^{IS}/0,04^{C})	832	1.29 [0.81-2.06]	0.29	0.42
rs12009 *HSPA5* (G/A)	G (0,44^{IS}/0,44^{C})	721	1.01 [0.82-1.24]	0.95	1.00	G (0,43^{IS}/0,41^{C})	834	1.07 [0.87-1.31]	0.53	0.56
rs430397 *HSPA5* (C/T)	T (0,08^{IS}/0,08^{C})	720	0.97 [0.67-1.40]	0.88	0.86	T (0,09^{IS}/0,09^{C})	825	0.92 [0.66-1.29]	0.63	0.56
rs753856 *HSPA6* (C/G)	G (0,10^{IS}/0,12^{C})	719	0.82 [0.58-1.16]	0.25	0.28	G (0,09^{IS}/0,12^{C})	829	**0.70 [0.50-0.98]**	**0.04**	**0.03**
rs1042665 *HSPA9* (T/C)	C (0,22^{IS}/0,20^{C})	715	1.13 [0.86-1.46]	0.38	0.29	C (0,23^{IS}/0,23^{C})	828	1.02 [0.80-1.29]	0.90	0.86
rs17155992 *HSPA14* (G/A)	A (0,09^{IS}/0,07^{C})	718	1.35 [0.90-2.01]	0.15	0.15	A (0,07^{IS}/0,08^{C})	832	0.94 [0.66-1.36]	0.75	0.86
rs706121 *BAG1* (C/T)	C (0,18^{IS}/0,13^{C})	705	**1.40 [1.04-1.89]**	**0.03**	**0.02**	C (0,13^{IS}/0,16^{C})	813	0.79 [0.59-1.04]	0.09	0.08
rs3778515 *BAG2* (T/G)	G (0,23^{IS}/0,25^{C})	674	0.92 [0.71-1.19]	0.51	0.48	G (0,26^{IS}/0,24^{C})	769	1.14 [0.90-1.44]	0.29	0.29
rs7749960 *BAG2* (C/A)	A (0,08^{IS}/0,13^{C})	716	**0.58 [0.41-0.82]**	**0.002**	**0.002**	A (0,11^{IS}/0,13^{C})	829	0.82 [0.60-1.11]	0.20	0.23
rs196336 *BAG3* (T/C)	T (0,43^{IS}/0,44^{C})	718	0.94 [0.75-1.16]	0.55	0.78	T (0,41^{IS}/0,40^{C})	832	1.01 [0.82-1.23]	0.93	0.86

rs196345<br>*BAG3*<br>(G/A)	A<br>(0,36^{IS}/0,33^{C})	674	1.15<br>[0.92-1.43]	0.23	0.37	A<br>(0,35^{IS}/0,36^{C})	755	0.96<br>[0.78-1.19]	0.73	1.00
rs196325<br>*BAG3*<br>(A/G)	A<br>(0,42^{IS}/0,44^{C})	719	0.92<br>[0.74-1.14]	0.44	0.41	A<br>(0,42^{IS}/0,41^{C})	835	1.06<br>[0.87-1.28]	0.57	0.78
rs2234962<br>*BAG3*<br>(T/C)	C<br>(0,21^{IS}/0,20^{C})	720	1.06<br>[0.82-1.37]	0.65	0.86	C<br>(0,21^{IS}/0,20^{C})	828	1.07<br>[0.84-1.36]	0.60	0.86
rs196329<br>*BAG3*<br>(A/G)	A<br>(0,27^{IS}/0,26^{C})	710	1.02<br>[0.81-1.29]	0.87	1.00	A<br>(0,25^{IS}/0,22^{C})	829	1.16<br>[0.93-1.46]	0.19	0.20
rs12682626<br>*BAG4*<br>(A/G)	G<br>(0,22^{IS}/0,23^{C})	717	0.97<br>[0.75-1.25]	0.82	0.78	G<br>(0,21^{IS}/0,21^{C})	833	0.99<br>[0.77-1.27]	0.96	1.00
rs11625397<br>*BAG5*<br>(C/T)	C<br>(0,34^{IS}/0,33^{C})	666	1.01<br>[0.79-1.28]	0.95	1.00	C<br>(0,33^{IS}/0,31^{C})	785	1.08<br>[0.86-1.35]	0.49	0.55
All calculations were performed relative to the minor alleles (Effect allele) with adjustment for age and smoking; 1 - odds ratio and 95% confidence interval; 2– p – value, 3 – p-value after permutation test. IS – patients; C – controls.										

Table S3. Results of the analysis of associations between HSP70 SNPs and IS risk in groups stratified by smoking status.

Genetic variant	Effect allele (frequency)	N	OR [95% CI][1]	*p*[2]	*p*$_{perm}$[3]	Effect allele (frequency)	N	OR [95% CI][1]	*p*[2]	*p*$_{perm}$[3]
	Smokers<br>(n=584: 387^{IS}; 197^{C})					Nonsmokers<br>(n=978: 381^{IS}; 597^{C})				
rs1043618<br>*HSPA1A*<br>(G/C)	C<br>(0,34^{IS}/0,37^{C})	577	0.89<br>[0.69-1.14]	0.36	0.34	C<br>(0,35^{IS}/0,36^{C})	967	0.97<br>[0.80-1.17]	0.73	0.73
rs2227956<br>*HSPA1L*<br>(G/A)	G<br>(0,17^{IS}/0,17^{C})	478	0.97<br>[0.67-1.41]	0.88	0.86	G<br>(0,13^{IS}/0,20^{C})	837	**0.60<br>[0.45-0.79]**	**0.0003**	**0.0002**
rs2075799<br>*HSPA1L*<br>(C/T)	T<br>(0,13^{IS}/0,12^{C})	577	1.14<br>[0.79-1.65]	0.48	0.56	T<br>(0,12^{IS}/0,11^{C})	961	1.16<br>[0.87-1.54]	0.32	0.33
rs1063391<br>*HSPA2*<br>(C/T)	C<br>(0,31^{IS}/0,29^{C})	572	1.08<br>[0.82-1.42]	0.59	0.67	C<br>(0,33^{IS}/0,35^{C})	955	0.93<br>[0.77-1.14]	0.50	0.40
rs12173082<br>*HSPA4*<br>(C/G)	G<br>(0,23^{IS}/0,23^{C})	575	0.95<br>[0.72-1.27]	0.74	0.78	G<br>(0,22^{IS}/0,23^{C})	966	0.91<br>[0.73-1.13]	0.39	0.29
rs13161158<br>*HSPA4*<br>(T/C)	C<br>(0,07^{IS}/0,07^{C})	583	0.93<br>[0.58-1.48]	0.75	1.00	C<br>(0,08^{IS}/0,06^{C})	975	1.36<br>[0.95-1.95]	0.09	0.11
rs4616886<br>*HSPA4*<br>(T/C)	C<br>(0,16^{IS}/0,15^{C})	538	1.05<br>[0.76-1.46]	0.76	0.86	C<br>(0,12^{IS}/0,13^{C})	923	0.92<br>[0.70-1.21]	0.56	0.86
rs11749966<br>*HSPA4*<br>(T/C)	C<br>(0,04^{IS}/0,04^{C})	581	1.15<br>[0.63-2.09]	0.65	0.86	C<br>(0,06^{IS}/0,05^{C})	971	1.35<br>[0.89-2.05]	0.15	0.12
rs12009<br>*HSPA5*<br>(G/A)	G<br>(0,44^{IS}/0,39^{C})	582	1.21<br>[0.94-1.54]	0.14	0.19	G<br>(0,43^{IS}/0,43^{C})	973	1.01<br>[0.84-1.21]	0.94	0.86
rs430397<br>*HSPA5*<br>(C/T)	T<br>(0,08^{IS}/0,08^{C})	580	0.95<br>[0.62-1.46]	0.81	0.86	T<br>(0,09^{IS}/0,09^{C})	965	0.98<br>[0.72-1.33]	0.90	1.00
rs753856<br>*HSPA6*<br>(C/G)	G<br>(0,08^{IS}/0,12^{C})	579	0.67<br>[0.44-1.01]	0.05	0.06	G<br>(0,11^{IS}/0,12^{C})	969	0.88<br>[0.65-1.18]	0.38	0.41

rs1042665 *HSPA9* (T/C)	C (0,25^{IS}/0,20^{C})	575	1.32 [0.97-1.79]	0.08	0.08	C (0,20^{IS}/0,22^{C})	968	0.88 [0.70-1.11]	0.29	0.34
rs17155992 *HSPA14* (G/A)	A (0,08^{IS}/0,06^{C})	578	1.36 [0.84-2.21]	0.21	0.22	A (0,08^{IS}/0,08^{C})	972	1.06 [0.76-1.47]	0.75	0.78
rs706121 *BAG1* (C/T)	C (0,17^{IS}/0,15^{C})	569	1.23 [0.87-1.73]	0.25	0.35	C (0,14^{IS}/0,15^{C})	949	0.90 [0.70-1.16]	0.41	0.48
rs3778515 *BAG2* (T/G)	G (0,23^{IS}/0,25^{C})	546	0.88 [0.66-1.18]	0.40	0.45	G (0,26^{IS}/0,24^{C})	897	1.12 [0.90-1.40]	0.31	0.27
rs7749960 *BAG2* (C/A)	A (0,08^{IS}/0,15^{C})	576	**0.50 [0.34-0.74]**	**0.001**	**0.001**	A (0,11^{IS}/0,13^{C})	969	0.83 [0.62-1.10]	0.19	0.19
rs196336 *BAG3* (T/C)	T (0,41^{IS}/0,44^{C})	581	0.88 [0.69-1.13]	0.32	0.52	T (0,42^{IS}/0,41^{C})	969	1.05 [0.87-1.27]	0.64	0.73
rs196345 *BAG3* (G/A)	A (0,35^{IS}/0,29^{C})	530	1.28 [0.98-1.66]	0.07	0.07	A (0,37^{IS}/0,37^{C})	899	1.00 [0.82-1.21]	0.97	1.00
rs196325 *BAG3* (A/G)	A (0,41^{IS}/0,42^{C})	581	0.95 [0.75-1.22]	0.70	0.78	A (0,43^{IS}/0,42^{C})	973	1.06 [0.88-1.28]	0.53	0.67
rs2234962 *BAG3* (T/C)	C (0,21^{IS}/0,17^{C})	577	1.30 [0.95-1.78]	0.10	0.09	C (0,21^{IS}/0,21^{C})	971	1.00 [0.80-1.25]	1.00	1.00
rs196329 *BAG3* (A/G)	A (0,26^{IS}/0,24^{C})	579	1.13 [0.85-1.49]	0.39	0.37	A (0,25^{IS}/0,24^{C})	960	1.10 [0.89-1.35]	0.39	0.48
rs12682626 *BAG4* (A/G)	G (0,22^{IS}/0,23^{C})	578	0.96 [0.72-1.28]	0.76	0.86	G (0,21^{IS}/0,21^{C})	972	0.98 [0.78-1.24]	0.87	1.00
rs11625397 *BAG5* (C/T)	C (0,32^{IS}/0,33^{C})	543	0.99 [0.76-1.31]	0.97	1.00	C (0,34^{IS}/0,32^{C})	908	1.12 [0.91-1.38]	0.29	0.25

All calculations were performed relative to the minor alleles (Effect allele) with adjustment for sex, age; 1 - odds ratio and 95% confidence interval; 2– *p* – value, 3 – *p*-value after permutation test. IS – patients; C – controls.

Table S4. Results of the analysis of associations between HSP70 SNPs and IS risk in groups stratified by physical activity, fruit/vegetable intake.

Genetic variant	Effect allele (frequency)	N	OR [95% CI][1]	p[2]	p_{perm}[3] (p_{bonf})	Effect allele (frequency)	N	OR [95% CI][1]	p[2]	p_{perm}[3] (p_{bonf})
	Low physical activity level (n=1084: 290^{IS}; 794^{C})					Normal physical activity level (n=974: 180^{IS}; 794^{C})				
rs1043618 *HSPA1A* (G/C)	C (0,36^{IS}/0,36^{C})	1078	0.98 [0.80-1.19]	0.80	0.73 (1.46)	C (0,36^{IS}/0,36^{C})	972	0.99 [0.78-1.25]	0.92	1.00 (2.00)
rs2227956 *HSPA1L* (G/A)	G (0,13^{IS}/0,19^{C})	911	**0.66 [0.49-0.88]**	**0.005**	**0.005 (0.01)**	G (0,15^{IS}/0,19^{C})	817	0.78 [0.55-1.10]	0.15	0.17 (0.34)
rs2075799 *HSPA1L* (C/T)	T (0,14^{IS}/0,11^{C})	1070	1.34 [1.02-1.78]	0.04	0.04 (0.08)	T (0,12^{IS}/0,11^{C})	960	1.13 [0.79-1.60]	0.50	1.00 (2.00)
rs1063391 *HSPA2* (C/T)	C (0,31^{IS}/0,33^{C})	1058	0.88 [0.72-1.09]	0.24	0.24 (0.48)	C (0,34^{IS}/0,33^{C})	951	1.01 [0.79-1.28]	0.95	1.00 (2.00)
rs12173082 *HSPA4* (C/G)	G (0,25^{IS}/0,23^{C})	1071	1.09 [0.87-1.35]	0.46	0.56 (1.12)	G (0,22^{IS}/0,23^{C})	962	0.92 [0.70-1.20]	0.53	0.46 (0.92)

rs13161158 *HSPA4* (T/C)	C (0,08^{IS}/0,06^{C})	1084	1.23 [0.85-1.78]	0.26	0.31 (0.62)	C (0,08^{IS}/0,06^{C})	973	1.38 [0.90-2.11]	0.14	0.12 (0.24)
rs4616886 *HSPA4* (T/C)	C (0,13^{IS}/0,14^{C})	1020	0.94 [0.70-1.25]	0.67	0.78 (1.56)	C (0,17^{IS}/0,14^{C})	923	1.28 [0.94-1.73]	0.11	0.10 (0.20)
rs11749966 *HSPA4* (T/C)	C (0,06^{IS}/0,04^{C})	1080	1.37 [0.90-2.08]	0.15	0.17 (0.34)	C (0,05^{IS}/0,04^{C})	969	1.13 [0.66-1.94]	0.65	0.73 (1.46)
rs12009 *HSPA5* (G/A)	G (0,42^{IS}/0,42^{C})	1081	1.00 [0.83-1.21]	0.99	1.00 (2.00)	G (0,45^{IS}/0,42^{C})	971	1.15 [0.92-1.44]	0.23	0.30 (0.60)
rs430397 *HSPA5* (C/T)	T (0,09^{IS}/0,09^{C})	1075	0.95 [0.68-1.33]	0.77	1.00 (2.00)	T (0,10^{IS}/0,09^{C})	966	1.07 [0.74-1.55]	0.73	0.86 (1.72)
rs753856 *HSPA6* (C/G)	G (0,10^{IS}/0,12^{C})	1073	0.85 [0.62-1.16]	0.30	0.38 (0.76)	G (0,08^{IS}/0,12^{C})	**964**	**0.60 [0.40-0.91]**	**0.02**	0.02 (0.04)
rs1042665 *HSPA9* (T/C)	C (0,23^{IS}/0,22^{C})	1076	1.09 [0.87-1.38]	0.46	0.46 (0.92)	C (0,22^{IS}/0,22^{C})	969	1.00 [0.76-1.31]	0.97	1.00 (2.00)
rs17155992 *HSPA14* (G/A)	A (0,07^{IS}/0,07^{C})	1077	1.02 [0.71-1.46]	0.93	1.00 (2.00)	A (0,07^{IS}/0,07^{C})	966	1.00 [0.66-1.53]	0.99	1.00 (2.00)
rs706121 *BAG1* (C/T)	C (0,15^{IS}/0,15^{C})	1042	0.98 [0.75-1.27]	0.86	0.73 (1.46)	C (0,13^{IS}/0,15^{C})	933	0.82 [0.59-1.14]	0.23	0.21 (0.42)
rs3778515 *BAG2* (T/G)	G (0,23^{IS}/0,24^{C})	1003	0.92 [0.73-1.18]	0.52	0.42 (0.84)	G (0,28^{IS}/0,24^{C})	900	1.24 [0.94-1.63]	0.13	0.10 (0.20)
rs7749960 *BAG2* (C/A)	A (0,09^{IS}/0,13^{C})	1074	**0.69 [0.50-0.95]**	**0.02**	**0.02 (0.04)**	A (0,11^{IS}/0,13^{C})	965	0.83 [0.58-1.19]	0.31	0.26 (0.52)
rs196336 *BAG3* (T/C)	T (0,42^{IS}/0,42^{C})	1082	1.03 [0.85-1.24]	0.80	0.86 (1.72)	T (0,39^{IS}/0,42^{C})	972	0.90 [0.71-1.13]	0.37	0.39 (0.78)
rs196345 *BAG3* (G/A)	A (0,35^{IS}/0,35^{C})	1010	0.99 [0.80-1.21]	0.89	0.86 (1.72)	A (0,36^{IS}/0,35^{C})	900	1.01 [0.79-1.30]	0.92	1.00 (2.00)
rs196325 *BAG3* (A/G)	A (0,43^{IS}/0,42^{C})	1078	1.05 [0.87-1.28]	0.59	0.73 (1.46)	A (0,41^{IS}/0,42^{C})	970	0.96 [0.77-1.20]	0.70	0.64 (1.28)
rs2234962 *BAG3* (T/C)	C (0,20^{IS}/0,20^{C})	1077	0.98 [0.78-1.24]	0.90	0.86 (1.72)	C (0,22^{IS}/0,20^{C})	966	1.11 [0.85-1.44]	0.46	0.39 (0.78)
rs196329 *BAG3* (A/G)	A (0,26^{IS}/0,24^{C})	1076	1.12 [0.90-1.40]	0.29	0.42 (0.84)	A (0,24^{IS}/0,24^{C})	972	1.03 [0.79-1.33]	0.84	0.86 (1.72)
rs12682626 *BAG4* (A/G)	G (0,20^{IS}/0,22^{C})	1080	0.91 [0.72-1.16]	0.46	0.63 (1.26)	G (0,22^{IS}/0,22^{C})	968	1.02 [0.77-1.36]	0.88	0.86 (1.72)
rs11625397 *BAG5* (C/T)	C (0,35^{IS}/0,32^{C})	1010	1.14 [0.92-1.41]	0.24	0.32 (0.64)	C (0,33^{IS}/0,32^{C})	973	1.06 [0.81-1.37]	0.68	0.64 (1.28)
		Low fruit/vegetable intake (n=1187: 393^{IS}; 794^{C})					Normal fruit/vegetable intake (n=1169: 375^{IS}; 794^{C})			
rs1043618 *HSPA1A* (G/C)	C (0,35^{IS}/0,36^{C})	1181	0.93 [0.78-1.12]	0.45	0.67 (1.34)	C (0,35^{IS}/0,36^{C})	1156	0.94 [0.78-1.12]	0.48	0.86 (1.72)
rs2227956 *HSPA1L* (G/A)	G (0,16^{IS}/0,19^{C})	998	0.78 [0.60-1.00]	0.05	0.04 (0.08)	G (0,14^{IS}/0,19^{C})	982	**0.69 [0.53-0.90]**	**0.006**	**0.005 (0.01)**

rs2075799 *HSPA1L* (C/T)	T (0,13^{IS}/0,11^{C})	1170	1.24 [0.95-1.60]	0.11	0.14 (0.28)	T (0,12^{IS}/0,11^{C})	1154	1.12 [0.86-1.47]	0.40	0.43 (0.86)
rs1063391 *HSPA2* (C/T)	C (0,32^{IS}/0,33^{C})	1159	0.92 [0.77-1.11]	0.40	0.59 (1.18)	C (0,32^{IS}/0,33^{C})	1143	0.96 [0.79-1.16]	0.67	0.86 (1.72)
rs12173082 *HSPA4* (C/G)	G (0,23^{IS}/0,23^{C})	1169	0.96 [0.79-1.18]	0.73	0.56 (1.12)	G (0,22^{IS}/0,23^{C})	1157	0.90 [0.73-1.11]	0.31	0.32 (0.64)
rs13161158 *HSPA4* (T/C)	C (0,08^{IS}/0,06^{C})	1186	1.34 [0.97-1.86]	0.08	0.09 (0.18)	C (0,06^{IS}/0,06^{C})	1166	1.04 [0.73-1.48]	0.84	0.86 (1.72)
rs4616886 *HSPA4* (T/C)	C (0,15^{IS}/0,14^{C})	1119	1.14 [0.90-1.45]	0.27	0.29 (0.58)	C (0,13^{IS}/0,14^{C})	1098	0.93 [0.72-1.21]	0.58	0.55 (1.10)
rs11749966 *HSPA4* (T/C)	C (0,05^{IS}/0,04^{C})	1183	1.15 [0.77-1.73]	0.50	0.52 (1.04)	C (0,05^{IS}/0,04^{C})	1160	1.25 [0.84-1.85]	0.27	0.21 (0.42)
rs12009 *HSPA5* (G/A)	G (0,44^{IS}/0,42^{C})	1180	1.06 [0.89-1.26]	0.52	0.56 (1.12)	G (0,43^{IS}/0,42^{C})	1167	1.05 [0.89-1.25]	0.56	0.52 (1.04)
rs430397 *HSPA5* (C/T)	T (0,09^{IS}/0,09^{C})	1177	0.96 [0.71-1.29]	0.78	0.73 (1.46)	T (0,08^{IS}/0,09^{C})	1159	0.90 [0.66-1.22]	0.50	0.48 (0.96)
rs753856 *HSPA6* (C/G)	G (0,09^{IS}/0,12^{C})	1176	**0.69 [0.51-0.93]**	**0.01**	**0.02 (0.04)**	G (0,10^{IS}/0,12^{C})	1158	0.85 [0.64-1.13]	0.27	0.36 (0.72)
rs1042665 *HSPA9* (T/C)	C (0,22^{IS}/0,22^{C})	1180	0.99 [0.80-1.22]	0.90	1.00 (2.00)	C (0,23^{IS}/0,22^{C})	1153	1.10 [0.89-1.36]	0.37	0.33 (0.66)
rs17155992 *HSPA14* (G/A)	A (0,09^{IS}/0,07^{C})	1176	1.23 [0.90-1.67]	0.19	0.19 (0.38)	A (0,07^{IS}/0,07^{C})	1161	1.03 [0.74-1.43]	0.88	0.86 (1.72)
rs706121 *BAG1* (C/T)	C (0,16^{IS}/0,15^{C})	1145	1.04 [0.82-1.30]	0.77	0.63 (1.26)	C (0,16^{IS}/0,15^{C})	1126	1.04 [0.82-1.31]	0.77	0.78 (1.56)
rs3778515 *BAG2* (T/G)	G (0,25^{IS}/0,24^{C})	1098	1.04 [0.84-1.28]	0.70	0.56 (1.12)	G (0,24^{IS}/0,24^{C})	1085	1.00 [0.80-1.23]	0.97	1.00 (2.00)
rs7749960 *BAG2* (C/A)	A (0,09^{IS}/0,13^{C})	1176	**0.62 [0.47-0.84]**	**0.002**	**0.001 (0.002)**	A (0,10^{IS}/0,13^{C})	1157	0.75 [0.57-0.99]	0.04	0.04 (0.08)
rs196336 *BAG3* (T/C)	T (0,39^{IS}/0,42^{C})	1185	0.90 [0.75-1.07]	0.22	0.27 (0.54)	T (0,44^{IS}/0,42^{C})	1157	1.11 [0.93-1.33]	0.24	0.32 (0.64)
rs196345 *BAG3* (G/A)	A (0,37^{IS}/0,35^{C})	1096	1.08 [0.89-1.29]	0.44	0.86 (1.72)	A (0,35^{IS}/0,35^{C})	1084	1.00 [0.83-1.21]	0.98	1.00 (2.00)
rs196325 *BAG3* (A/G)	A (0,42^{IS}/0,42^{C})	1180	0.98 [0.83-1.17]	0.86	1.00 (2.00)	A (0,43^{IS}/0,42^{C})	1165	1.03 [0.86-1.22]	0.78	0.73 (1.46)
rs2234962 *BAG3* (T/C)	C (0,22^{IS}/0,20^{C})	1179	1.11 [0.91-1.37]	0.31	0.36 (0.72)	C (0,20^{IS}/0,20^{C})	1159	1.01 [0.82-1.25]	0.91	1.00 (2.00)
rs196329 *BAG3* (A/G)	A (0,24^{IS}/0,24^{C})	1179	1.01 [0.82-1.23]	0.96	1.00 (2.00)	A (0,28^{IS}/0,24^{C})	1154	**1.25 [1.03-1.52]**	**0.03**	**0.02 (0.04)**
rs12682626 *BAG4* (A/G)	G (0,22^{IS}/0,22^{C})	1179	0.99 [0.80-1.22]	0.90	1.00 (2.00)	G (0,22^{IS}/0,22^{C})	1162	1.00 [0.80-1.24]	0.98	1.00 (2.00)
rs11625397 *BAG5* (C/T)	C (0,32^{IS}/0,32^{C})	1105	1.00 [0.82-1.22]	1.00	1.00 (2.00)	C (0,35^{IS}/0,32^{C})	1092	1.12 [0.92-1.37]	0.26	0.33 (0.66)

All calculations were performed relative to the minor alleles (Effect allele) with adjustment for sex, age and smoking; 1 - odds ratio and 95% confidence interval; 2– *p* – value, 3 – *p*-value after permutation test. IS – patients; C – controls.

Table S5. Sex-stratified associations between HSP70-related genetic variants and ischemic stroke risk and assessment of between-sex heterogeneity.

SNP	Gene	Effect allele	OR Men [95% CI]	p_g0	OR Women [95% CI]	p_g1	p_ interaction	p_het_Q	FDR (p_het)	I2
rs4616886	*HSPA4*	C	0.76 [0.56-1.02]	0.066	1.17 [0.85-1.60]	0.339	0.034	0.051	0.67	74%
rs706121	*BAG1*	C	1.28 [0.94-1.75]	0.113	0.87 [0.65-1.18]	0.384	0.072	0.082	0.67	67%
rs2075799	*HSPA1L*	T	1.51 [1.05-2.17]	0.026	0.99 [0.72-1.36]	0.954	0.081	0.087	0.67	66%
rs196325	*BAG3*	A	0.91 [0.73-1.14]	0.406	1.12 [0.91-1.38]	0.286	0.163	0.182	0.849	44%
rs7749960	*BAG2*	A	0.59 [0.41-0.84]	0.004	0.82 [0.59-1.13]	0.223	0.164	0.187	0.849	43%
rs1063391	*HSPA2*	C	1.12 [0.87-1.44]	0.362	0.91 [0.73-1.15]	0.435	0.193	0.23	0.849	31%
rs17155992	*HSPA14*	A	1.31 [0.87-1.98]	0.2	0.94 [0.64-1.40]	0.768	0.299	0.258	0.849	22%
rs3778515	*BAG2*	G	0.93 [0.72-1.21]	0.613	1.13 [0.88-1.46]	0.339	0.312	0.304	0.873	5%
rs12009	*HSPA5*	G	1.02 [0.83-1.27]	0.843	1.14 [0.92-1.42]	0.237	0.541	0.478	0.96	0%
rs196345	*BAG3*	A	1.17 [0.93-1.47]	0.171	1.05 [0.83-1.32]	0.689	0.388	0.493	0.96	0%
rs12173082	*HSPA4*	G	0.92 [0.71-1.18]	0.503	1.01 [0.79-1.30]	0.932	0.71	0.592	0.96	0%
rs2234962	*BAG3*	C	1.04 [0.80-1.35]	0.78	1.15 [0.88-1.49]	0.303	0.595	0.599	0.96	0%
rs11749966	*HSPA4*	C	1.25 [0.76-2.06]	0.371	1.48 [0.89-2.45]	0.127	0.864	0.647	0.96	0%
rs753856	*HSPA6*	G	0.83 [0.58-1.18]	0.293	0.75 [0.52-1.07]	0.115	0.711	0.707	0.96	0%
rs1043618	*HSPA1A*	C	1.00 [0.79-1.26]	0.993	0.94 [0.76-1.17]	0.594	0.663	0.709	0.96	0%
rs2227956	*HSPA1L*	G	0.63 [0.46-0.87]	0.005	0.69 [0.49-0.95]	0.025	0.644	0.743	0.96	0%
rs11625397	*BAG5*	C	1.00 [0.78-1.28]	0.991	1.06 [0.83-1.35]	0.656	0.713	0.749	0.96	0%
rs12682626	*BAG4*	G	0.97 [0.75-1.26]	0.803	1.02 [0.78-1.34]	0.883	0.859	0.78	0.96	0%
rs13161158	*HSPA4*	C	1.22 [0.78-1.91]	0.388	1.13 [0.75-1.70]	0.561	0.88	0.805	0.96	0%
rs196329	*BAG3*	A	1.06 [0.83-1.35]	0.652	1.09 [0.85-1.40]	0.483	0.925	0.857	0.96	0%
rs430397	*HSPA5*	T	1.00 [0.68-1.45]	0.981	0.96 [0.67-1.38]	0.814	0.882	0.885	0.96	0%
rs1042665	*HSPA9*	C	1.06 [0.81-1.39]	0.666	1.04 [0.80-1.35]	0.764	0.914	0.919	0.96	0%
rs196336	*BAG3*	T	0.97 [0.77-1.21]	0.775	0.96 [0.78-1.20]	0.742	0.991	0.98	0.98	0%

Effect allele, allele for which the association was estimated; OR, odds ratio calculated under an additive genetic model with adjustment for age and smoking status; CI, confidence interval; p_g0 and p_g1, nominal association p-values for men and women, respectively; p_interaction, p-value for the genotype × sex interaction; p_het_Q, p-value from Cochran's Q test comparing the sex-specific effect estimates; FDR (p_het), Benjamini–Hochberg-adjusted heterogeneity p-value, accounting for multiple testing across the 23 investigated SNPs; I^2, heterogeneity statistic expressing the proportion of variability in effect estimates attributable to between-group heterogeneity, expressed as a percentage.

Table S6. Smoking-stratified associations between HSP70-related genetic variants and ischemic stroke risk and assessment of between-group heterogeneity.

SNP	Gene	Effect allele	OR Nonsmokers [95% CI]	p_g0	OR Smokers [95% CI]	p_g1	p_ interaction	p_het_Q	FDR (p_het)	I2
rs2227956	*HSPA1L*	G	0.54 [0.40-0.73]	0	0.93 [0.63-1.37]	0.724	0.034	0.029	0.671	79%
rs7749960	*BAG2*	A	0.83 [0.62-1.12]	0.22	0.52 [0.35-0.78]	0.001	0.071	0.068	0.671	70%
rs1042665	*HSPA9*	C	0.92 [0.73-1.17]	0.497	1.30 [0.95-1.78]	0.103	0.083	0.087	0.671	66%
rs196345	*BAG3*	A	1.03 [0.84-1.26]	0.785	1.27 [0.97-1.65]	0.084	0.237	0.226	0.759	32%
rs12009	*HSPA5*	G	1.00 [0.83-1.21]	0.995	1.20 [0.94-1.54]	0.149	0.267	0.255	0.759	23%
rs3778515	*BAG2*	G	1.12 [0.89-1.41]	0.335	0.90 [0.67-1.21]	0.489	0.267	0.256	0.759	22%

rs753856	*HSPA6*	G	0.86 [0.63-1.17]	0.349	0.65 [0.43-0.99]	0.047	0.267	0.29	0.759	11%
rs13161158	*HSPA4*	C	1.32 [0.90-1.92]	0.153	0.96 [0.59-1.54]	0.857	0.315	0.304	0.759	5%
rs2234962	*BAG3*	C	1.01 [0.81-1.27]	0.91	1.24 [0.90-1.70]	0.189	0.339	0.317	0.759	0%
rs196336	*BAG3*	T	1.03 [0.84-1.25]	0.791	0.89 [0.69-1.14]	0.346	0.358	0.365	0.759	0%
rs706121	*BAG1*	C	0.97 [0.74-1.26]	0.797	1.17 [0.82-1.66]	0.384	0.418	0.396	0.759	0%
rs17155992	*HSPA14*	A	1.01 [0.71-1.43]	0.967	1.29 [0.79-2.11]	0.303	0.447	0.415	0.759	0%
rs1063391	*HSPA2*	C	0.96 [0.78-1.18]	0.694	1.10 [0.83-1.46]	0.489	0.394	0.429	0.759	0%
rs11625397	*BAG5*	C	1.10 [0.88-1.37]	0.411	0.96 [0.73-1.27]	0.795	0.46	0.476	0.781	0%
rs1043618	*HSPA1A*	C	1.01 [0.83-1.23]	0.922	0.91 [0.71-1.18]	0.483	0.577	0.539	0.826	0%
rs196325	*BAG3*	A	1.04 [0.86-1.26]	0.712	0.95 [0.74-1.22]	0.694	0.572	0.591	0.848	0%
rs4616886	*HSPA4*	C	0.89 [0.67-1.18]	0.419	0.99 [0.71-1.39]	0.965	0.656	0.627	0.848	0%
rs12173082	*HSPA4*	G	0.94 [0.75-1.17]	0.567	1.01 [0.75-1.35]	0.953	0.66	0.694	0.887	0%
rs11749966	*HSPA4*	C	1.44 [0.94-2.22]	0.094	1.28 [0.69-2.35]	0.433	0.771	0.744	0.9	0%
rs2075799	*HSPA1L*	T	1.22 [0.91-1.64]	0.194	1.15 [0.79-1.68]	0.465	0.86	0.822	0.946	0%
rs430397	*HSPA5*	T	0.98 [0.71-1.35]	0.917	0.95 [0.61-1.47]	0.806	0.89	0.891	0.96	0%
rs12682626	*BAG4*	G	1.00 [0.78-1.27]	0.98	0.98 [0.73-1.31]	0.881	0.926	0.92	0.96	0%
rs196329	*BAG3*	A	1.09 [0.88-1.36]	0.417	1.10 [0.83-1.47]	0.501	0.975	0.96	0.96	0%

Effect allele, allele for which the association was estimated; OR, odds ratio calculated under an additive genetic model with adjustment for age and sex; CI, confidence interval; p_g0 and p_g1, nominal association p-values for nonsmokers and smokers, respectively; p_interaction, p-value for the genotype × smoking status interaction; p_het_Q, p-value from Cochran's Q test comparing the smoking-specific effect estimates; FDR (p_het), Benjamini–Hochberg-adjusted heterogeneity p-value, accounting for multiple testing across the 23 investigated SNPs; I^2, heterogeneity statistic expressing the proportion of variability in effect estimates attributable to between-group heterogeneity, expressed as a percentage.

Table S7. STRING functional associations and confidence scores for selected HSP70 family proteins and BAG co-chaperones

Protein encoding an IS-related gene	PPI-proteins	Functions of PPI-proteins	*						Score
			1	2	3	4	5	6	
**HSPA1L**	HSP90AA1	HSP90α is a molecular chaperone that maintains protein stability and regulates the function of key proteins involved in cell-cycle control and signal transduction. Its activity depends on an ATPase-driven cycle and is modulated by various co-chaperones.			•	•	•	•	0.997
	STIP1	Stress-induced-phosphoprotein 1; Acts as a co-chaperone for HSP90AA1. Mediates the association of the molecular chaperones HSPA8/HSC70 and HSP90			•	•	•	•	0.835
	NR3C1	Glucocorticoid receptor (GR) is a ligand-activated transcription factor that regulates gene expression involved in					•	•	0.540

		inflammation, cell proliferation, and differentiation. It acts through glucocorticoid response elements and modulates other transcription factors, thereby playing a key role in the cellular response to glucocorticoids.							
	DNAJB1	DNAJ homolog subfamily B member 1 (DNAJB1) is an HSP70 co-chaperone that stimulates HSP70 ATPase activity and promotes protein folding. It also contributes to the regulation of the heat-shock response by suppressing HSF1 activity during recovery.		•	•	•	•	•	0.992
	DNAJA1	DnaJ homolog subfamily A member 1 (DNAJA1) is an HSP70 co-chaperone that stimulates ATP hydrolysis and facilitates protein transport, particularly into mitochondria. It also regulates stress-induced BAX translocation and apoptosis, contributing to cellular stress responses.		•	•	•	•	•	0.976
	DNAJB4	DnaJ homolog subfamily B member 4; Probable chaperone. Stimulates ATP hydrolysis and the folding of unfolded proteins mediated by HSPA1A/B (in vitro).		•	•	•	•	•	0.986
	DNAJA2	DnaJ homolog subfamily A member 2; Co-chaperone of Hsc70. Stimulates ATP hydrolysis and the folding of unfolded proteins mediated by HSPA1A/B (in vitro).		•	•	•	•	•	0.937
	HSP90AB1	HSP90β is a molecular chaperone that promotes the maturation, stabilization, and functional regulation of client proteins, including those involved in cell-cycle control and signal transduction. Its chaperone activity is ATP-dependent and regulated by co-chaperones, which facilitate client protein recognition and folding.			•	•	•	•	0.996
	DNAJC7	DNAJC7 is a co-chaperone that regulates HSP70 and HSP90			•	•	•	•	0.993

		activity during steroid receptor folding, including glucocorticoid and progesterone receptors. It may also function as a recycling chaperone, facilitating the re-entry of substrates into the chaperone cycle when further folding is required.							
	TLR2	Toll-like receptor 2 (TLR2) is a key innate immune receptor that recognizes bacterial and fungal components, as well as endogenous danger signals. In cooperation with TLR1 or TLR6, it activates MyD88/TRAF6–NF-κB signaling, inducing pro-inflammatory cytokine production and the innate immune response.				•	•	•	0.904
**HSPA4**	STUB1	E3 ubiquitin-protein ligase CHIP (STUB1) is a co-chaperone of HSP70/HSP90 that directs misfolded proteins to the ubiquitin–proteasome system for degradation. It plays a central role in protein quality control and cellular stress responses and also regulates key signaling pathways involved in apoptosis, cell-cycle control, and DNA repair.			•	•		•	0.999
	DNAJB1	DNAJ homolog subfamily B member 1 (DNAJB1) is an HSP70 co-chaperone that stimulates HSP70 ATPase activity and promotes protein folding. It also contributes to the regulation of the heat-shock response by suppressing HSF1 activity during recovery.		•	•	•	•	•	0.999
	HSP90AA1	HSP90α is a molecular chaperone that maintains protein stability and regulates the function of key proteins involved in cell-cycle control and signal transduction. Its activity depends on an ATPase-driven cycle and is modulated by various co-chaperones.			•	•	•	•	0.999

	STIP1	Stress-induced-phosphoprotein 1; Acts as a co-chaperone for HSP90AA1. Mediates the association of the molecular chaperones HSPA8/HSC70 and HSP90.			•	•		•	0.999
	BAG3	BAG3 is an HSP70/HSC70 co-chaperone that functions as a nucleotide-exchange factor, promoting ADP release and subsequent release of client proteins. It also contributes to cell survival through anti-apoptotic activity and regulates HSF1 nucleocytoplasmic transport.			•	•	•	•	0.999
	HSP90AB1	HSP90β is a molecular chaperone that promotes the maturation, stabilization, and functional regulation of client proteins, including those involved in cell-cycle control and signal transduction. Its chaperone activity is ATP-dependent and regulated by co-chaperones, which facilitate client protein recognition and folding.			•	•	•	•	0.999
	HSPA8	HSPA8/Hsc70 is a molecular chaperone that plays a central role in protein quality control, folding, refolding, and degradation of misfolded proteins. Its activity is driven by the ATP–ADP cycle and regulated by co-chaperones, enabling substrate binding and release. HSPA8 also participates in clathrin-mediated endocytosis and chaperone-mediated autophagy, contributing to cellular stress protection.		•	•	•	•	•	0.999
	BAG1	BAG1 is an HSP70/HSC70 co-chaperone and nucleotide-exchange factor (NEF) that promotes ADP release and client protein dissociation from HSP70. It supports protein quality control and glucocorticoid receptor maturation and exerts anti-			•	•	•	•	0.999

		apoptotic effects, contributing to cell survival.							
	FKBP4	FKBP4 is an immunophilin and HSP90 co-chaperone that facilitates the intracellular trafficking and nuclear translocation of steroid hormone receptors. Its PPIase activity also regulates microtubule dynamics and neuronal function, linking FKBP4 to steroid receptor signaling and cellular transport.			•	•		•	0.900
	HSPBP1	Hsp70-binding protein 1; Inhibits HSPA1A chaperone activity by changing the conformation of the ATP-binding domain of HSPA1A and interfering with ATP binding. Interferes with ubiquitination mediated by STUB1 and inhibits chaperone-assisted degradation of immature CFTR.			•	•		•	0.978
**HSPA6**	DNAJB1	DNAJ homolog subfamily B member 1 (DNAJB1) is an HSP70 co-chaperone that stimulates HSP70 ATPase activity and promotes protein folding. It also contributes to the regulation of the heat-shock response by suppressing HSF1 activity during recovery.		•	•	•	•	•	0.992
	HSPA1B	HSPA1A (Hsp70-1A) is a stress-inducible molecular chaperone that plays a central role in protein quality control, promoting protein folding, refolding, and degradation of damaged proteins. Its ATP-dependent activity is regulated by co-chaperones and contributes to cellular stress protection and regulation of apoptosis.			•	•	•	•	0.999
	DNAJB4	DnaJ homolog subfamily B member 4; Probable chaperone. Stimulates ATP hydrolysis and the folding of unfolded proteins mediated by HSPA1A/B (in vitro).		•	•	•	•	•	0.942

	TLR2	Toll-like receptor 2 (TLR2) is a key innate immune receptor that recognizes bacterial and fungal components, as well as endogenous danger signals. In cooperation with TLR1 or TLR6, it activates MyD88/TRAF6–NF-κB signaling, inducing pro-inflammatory cytokine production and the innate immune response.			•	•	•	•	0.922
	HSPA4	Heat shock protein family A member 4; Belongs to the heat shock protein 70 family.		•	•	•	•	•	0.999
	MAPK8	MAPK8 (JNK1) is a stress-activated serine/threonine kinase that mediates cellular responses to inflammation, oxidative stress, DNA damage, and other stress stimuli. It regulates transcription factors and apoptotic proteins, thereby controlling apoptosis, cell proliferation, and immune responses.			•		•	•	0.909
	MAPK9	MAPK9 (JNK2) is a stress-activated serine/threonine kinase that mediates cellular responses to inflammatory and stress signals. It regulates transcription factors and apoptotic proteins, thereby influencing immune responses, apoptosis, cell proliferation, and inflammatory processes.			•		•	•	0.906
	DNAJA1	DnaJ homolog subfamily A member 1 (DNAJA1) is an HSP70 co-chaperone that stimulates ATP hydrolysis and facilitates protein transport, particularly into mitochondria. It also regulates stress-induced BAX translocation and apoptosis, contributing to cellular stress responses.		•	•	•	•	•	0.882
	DNAJB6	DnaJ homolog subfamily B member 6; Plays an indispensable role in the organization of KRT8/KRT18 filaments. Acts as an endogenous molecular		•	•	•	•	•	0.897

		chaperone for neuronal proteins including huntingtin. Suppresses aggregation and toxicity of polyglutamine-containing, aggregation-prone proteins. Isoform B but not isoform A inhibits huntingtin aggregation. Has a stimulatory effect on the ATPase activity of HSP70 in a dose-dependent and time-dependent manner and hence acts as a co-chaperone of HSP70. Also reduces cellular toxicity and caspase-3 activity.							
	HSP90AB1	HSP90β is a molecular chaperone that promotes the maturation, stabilization, and functional regulation of client proteins, including those involved in cell-cycle control and signal transduction. Its chaperone activity is ATP-dependent and regulated by co-chaperones, which facilitate client protein recognition and folding.			•	•	•	•	0.999
**BAG1**	HSPA4	Heat shock protein family A member 4; Belongs to the heat shock protein 70 family.			•	•	•	•	0.999
	HSPA8	HSPA8/Hsc70 is a molecular chaperone that plays a central role in protein quality control, folding, refolding, and degradation of misfolded proteins. Its activity is driven by the ATP–ADP cycle and regulated by co-chaperones, enabling substrate binding and release. HSPA8 also participates in clathrin-mediated endocytosis and chaperone-mediated autophagy, contributing to cellular stress protection.			•	•	•	•	0.999
	BAG2	BAG family molecular chaperone regulator 2; Co-chaperone for HSP70 and HSC70 chaperone proteins. Acts as a nucleotide-exchange factor (NEF) promoting the release of ADP from the HSP70 and HSC70 proteins					•	•	0.995

		thereby triggering client/substrate protein release.							
	BAG3	BAG3 is an HSP70/HSC70 co-chaperone that functions as a nucleotide-exchange factor, promoting ADP release and subsequent release of client proteins. It also contributes to cell survival through anti-apoptotic activity and regulates HSF1 nucleocytoplasmic transport.			•	•	•	•	0.969
	BCL2	Apoptosis regulator Bcl-2; Suppresses apoptosis in a variety of cell systems including factor-dependent lymphohematopoietic and neural cells. Regulates cell death by controlling the mitochondrial membrane permeability. Appears to function in a feedback loop system with caspases. Inhibits caspase activity either by preventing the release of cytochrome c from the mitochondria and/or by binding to the apoptosis-activating factor (APAF-1). May attenuate inflammation by impairing NLRP1-inflammasome activation, hence CASP1 activation and IL1B release.			•	•		•	0.922
	DNAJB1	DNAJ homolog subfamily B member 1 (DNAJB1) is an HSP70 co-chaperone that stimulates HSP70 ATPase activity and promotes protein folding. It also contributes to the regulation of the heat-shock response by suppressing HSF1 activity during recovery.			•	•		•	0.869
	HSPBP1	Hsp70-binding protein 1; Inhibits HSPA1A chaperone activity by changing the conformation of the ATP-binding domain of HSPA1A and interfering with ATP binding. Interferes with ubiquitination mediated by STUB1 and inhibits chaperone-			•		•	•	0.965

		assisted degradation of immature CFTR.							
	HSPA1B	HSPA1A (Hsp70-1A) is a stress-inducible molecular chaperone that plays a central role in protein quality control, promoting protein folding, refolding, and degradation of damaged proteins. Its ATP-dependent activity is regulated by co-chaperones and contributes to cellular stress protection and regulation of apoptosis.				•	•	•	0.970
	STUB1	E3 ubiquitin-protein ligase CHIP (STUB1) is a co-chaperone of HSP70/HSP90 that directs misfolded proteins to the ubiquitin–proteasome system for degradation. It plays a central role in protein quality control and cellular stress responses and also regulates key signaling pathways involved in apoptosis, cell-cycle control, and DNA repair.			•	•		•	0.999
	HSPH1	Heat shock protein 105 kDa; Acts as a nucleotide-exchange factor (NEF) for chaperone proteins HSPA1A and HSPA1B, promoting the release of ADP from HSPA1A/B thereby triggering client/substrate protein release. Prevents the aggregation of denatured proteins in cells under severe stress, on which the ATP levels decrease markedly. Inhibits HSPA8/HSC70 ATPase and chaperone activities.			•		•	•	0.934
**BAG2**	HSPA8	HSPA8/Hsc70 is a molecular chaperone that plays a central role in protein quality control, folding, refolding, and degradation of misfolded proteins. Its activity is driven by the ATP–ADP cycle and regulated by co-chaperones, enabling substrate binding and release. HSPA8 also participates in clathrin-			•	•	•	•	0.999

		mediated endocytosis and chaperone-mediated autophagy, contributing to cellular stress protection.							
	STUB1	E3 ubiquitin-protein ligase CHIP (STUB1) is a co-chaperone of HSP70/HSP90 that directs misfolded proteins to the ubiquitin–proteasome system for degradation. It plays a central role in protein quality control and cellular stress responses and also regulates key signaling pathways involved in apoptosis, cell-cycle control, and DNA repair.			•	•	•	•	0.999
	HSPA4	Heat shock protein family A member 4; Belongs to the heat shock protein 70 family.			•	•	•	•	0.986
	BAG1	BAG1 is an HSP70/HSC70 co-chaperone and nucleotide-exchange factor (NEF) that promotes ADP release and client protein dissociation from HSP70. It supports protein quality control and glucocorticoid receptor maturation and exerts anti-apoptotic effects, contributing to cell survival.					•	•	0.995
	HSPH1	Heat shock protein 105 kDa; Acts as a nucleotide-exchange factor (NEF) for chaperone proteins HSPA1A and HSPA1B, promoting the release of ADP from HSPA1A/B thereby triggering client/substrate protein release. Prevents the aggregation of denatured proteins in cells under severe stress, on which the ATP levels decrease markedly. Inhibits HSPA8/HSC70 ATPase and chaperone activities.			•		•	•	0.947
	BAG3	BAG3 is an HSP70/HSC70 co-chaperone that functions as a nucleotide-exchange factor, promoting ADP release and subsequent release of client proteins. It also contributes to cell survival through anti-			•	•	•	•	0.994

		apoptotic activity and regulates HSF1 nucleocytoplasmic transport.							
	HSPBP1	Hsp70-binding protein 1; Inhibits HSPA1A chaperone activity by changing the conformation of the ATP-binding domain of HSPA1A and interfering with ATP binding. Interferes with ubiquitination mediated by STUB1 and inhibits chaperone-assisted degradation of immature CFTR.			•	•		•	0.956
	BAG5	BAG family molecular chaperone regulator 5; Inhibits both auto-ubiquitination of PRKN and ubiquitination of target proteins by PRKN. May function as a nucleotide exchange factor for HSP/HSP70, promoting ADP release, and activating Hsp70-mediated refolding.			•	•	•	•	0.969
	HSPA4L	Heat shock 70 kDa protein 4L; Possesses chaperone activity in vitro where it inhibits aggregation of citrate synthase; Belongs to the heat shock protein 70 family.			•		•	•	0.917
	SGTA	SGTA is a co-chaperone that binds misfolded and hydrophobic proteins, directing them either to the endoplasmic reticulum for membrane insertion or to the proteasome for degradation. It also regulates steroid receptor stability and trafficking, including androgen and glucocorticoid receptors, contributing to protein quality control and cellular stress responses.			•	•		•	0.533
**BAG3**	STUB1	E3 ubiquitin-protein ligase CHIP (STUB1) is a co-chaperone of HSP70/HSP90 that directs misfolded proteins to the ubiquitin–proteasome system for degradation. It plays a central role in protein quality control and cellular stress			•	•	•	•	0.999

		responses and also regulates key signaling pathways involved in apoptosis, cell-cycle control, and DNA repair.								
	HSPB1	Heat shock protein beta-1; Small heat shock protein which functions as a molecular chaperone probably maintaining denatured proteins in a folding- competent state. Plays a role in stress resistance and actin organization. Through its molecular chaperone activity may regulate numerous biological processes including the phosphorylation and the axonal transport of neurofilament proteins.			•	•		•	0.995
	HSPB8	Heat shock protein beta-8; Displays temperature-dependent chaperone activity; Belongs to the small heat shock protein (HSP20) family.			•	•	•	•	0.999
	HSPA4	Heat shock protein family A member 4; Belongs to the heat shock protein 70 family.			•	•	•	•	0.999
	HSPA8	HSPA8/Hsc70 is a molecular chaperone that plays a central role in protein quality control, folding, refolding, and degradation of misfolded proteins. Its activity is driven by the ATP–ADP cycle and regulated by co-chaperones, enabling substrate binding and release. HSPA8 also participates in clathrin-mediated endocytosis and chaperone-mediated autophagy, contributing to cellular stress protection.			•	•	•	•	0.999
	BCL2	BCL2 is a key anti-apoptotic protein that prevents mitochondrial membrane permeabilization and cytochrome c release, thereby inhibiting caspase activation and programmed cell death. It may also modulate inflammatory responses by suppressing inflammasome activation.				•		•	0.741

	HSPA1B	HSPA1A (Hsp70-1A) is a stress-inducible molecular chaperone that plays a central role in protein quality control, promoting protein folding, refolding, and degradation of damaged proteins. Its ATP-dependent activity is regulated by co-chaperones and contributes to cellular stress protection and regulation of apoptosis.			•	•	•	•	0.999
	BAG2	BAG family molecular chaperone regulator 2; Co-chaperone for HSP70 and HSC70 chaperone proteins. Acts as a nucleotide-exchange factor (NEF) promoting the release of ADP from the HSP70 and HSC70 proteins thereby triggering client/substrate protein release.			•	•	•	•	0.994
	BAG1	BAG1 is an HSP70/HSC70 co-chaperone and nucleotide-exchange factor (NEF) that promotes ADP release and client protein dissociation from HSP70. It supports protein quality control and glucocorticoid receptor maturation and exerts anti-apoptotic effects, contributing to cell survival.			•	•	•	•	0.969
	HSP90AB1	HSP90β is a molecular chaperone that promotes the maturation, stabilization, and functional regulation of client proteins, including those involved in cell-cycle control and signal transduction. Its chaperone activity is ATP-dependent and regulated by co-chaperones, which facilitate client protein recognition and folding.			•	•	•	•	0.981

Data from the STRING database is shown.
* - Proven interaction mechanisms:
1 –Neighborhood; 2 – Cooccurence; 3 – Coexpression; 4 – Experiments; 5 – Databases; 6 - Textmining

Table S8. Relationship between HSP70 SNPs and cis-eQTL-mediated expression levels of genes in whole blood (according to browser eQTLGene; https://www.eqtlgen.org/) (accessed on 10 May 2026).

SNP	Effect allele	Gene expressed	Z-score	P-value	FDR
rs2227956 *HSPA1L* *(G/A)*	G	*HLA-DRB5*	↑(56.8344)	3.27×10^{-310}	0
		HLA-C	↑(30.2641)	3.41×10^{-201}	0
		HLA-B	↑(22.4426)	1.52×10^{-111}	0
		C4B	↓(-22.1215)	1.96×10^{-108}	0
		MICB	↓(-22.0020)	2.76×10^{-107}	0
		HLA-DQB1-AS1	↑(21.9540)	7.93×10^{-107}	0
		DDAH2	↑(21.2499)	3.31×10^{-100}	0
		C4A	↑(18.0613)	6.44×10^{-73}	0
		LY6G5B	↑(16.2978)	1.03×10^{-59}	0
		LY6G5C	↑(14.1572)	1.69×10^{-45}	0
		HLA-DQB1	↑(13.8356)	1.56×10^{-43}	0
		HCG27	↓(-12.6648)	9.24×10^{-37}	0
		ATP6V1G2	↓(-11.6773)	1.66×10^{-31}	0
		CYP21A2	↓(-11.5845)	4.93×10^{-31}	0
		HSPA1B	↑(11.3398)	8.35×10^{-30}	0
		SKIV2L	↓(-10.8241)	2.64×10^{-27}	0
		PSORS1C3	↓(-10.3911)	2.72×10^{-25}	0
		POU5F1	↓(-9.7990)	1.14×10^{-22}	0
		XXbac-BPG248L24.11	↓(-9.7786)	1.39×10^{-22}	0
		HLA-DRB1	↑(9.7742)	1.45×10^{-22}	0
		TNF	↑(9.7398)	2.04×10^{-22}	0
		TCF19	↓(-9.6018)	7.86×10^{-22}	0
		HLA-DQA1	↑(9.2447)	2.36×10^{-20}	0
		HLA-DQA2	↓(-9.2332)	2.62×10^{-20}	0
		TNXB	↓(-8.7422)	2.28×10^{-18}	0
		HLA-S	↓(-8.0495)	8.31×10^{-16}	0
		NCR3	↑(7.9738)	1.54×10^{-15}	0
		HLA-DRA	↓(-7.8730)	3.46×10^{-15}	0
		HLA-DRB6	↓(-7.7774)	7.40×10^{-15}	0
		HLA-DQB2	↓(-7.5211)	5.43×10^{-14}	0
		VWA7	↑(7.3172)	2.54×10^{-13}	0
		DDX39B	↑(7.2866)	3.18×10^{-13}	0
		PPT2	↑(6.6570)	2.80×10^{-11}	0
		ATF6B	↓(-6.3565)	2.06×10^{-10}	0
		DDR1	↓(-6.2057)	5.44×10^{-10}	0
		LTA	↑(6.1267)	8.98×10^{-10}	1.32×10^{-5}
		HCG22	↑(5.8694)	4.37×10^{-9}	3.24×10^{-5}
		LY6G6C	↑(5.5432)	2.97×10^{-9}	1.21×10^{-4}
		BAG6	↓(-5.4495)	5.05×10^{-8}	1.77×10^{-4}
		LTB	↑(5.4237)	5.84×10^{-8}	2.15×10^{-4}
		EGFL8	↑(5.3749)	7.67×10^{-8}	2.71×10^{-4}
		AGER	↓(-5.2160)	1.83×10^{-8}	5.75×10^{-4}
		AGPAT1	↑(5.1369)	2.79×10^{-7}	8.65×10^{-4}

		CCHCR1	↓(-4.9901)	6.03×10^{-7}	1.82×10^{-3}
		NOTCH4	↑(4.9700)	6.70×10^{-7}	1.95×10^{-3}
		MICA	↓(-4.8531)	1.22×10^{-6}	3.44×10^{-3}
		RNF5	↑(4.6301)	3.66×10^{-6}	9.82×10^{-3}
		EHMT2	↑(4.6194)	3.85×10^{-6}	1.03×10^{-2}
		VARS2	↓(-4.3923)	1.12×10^{-5}	2.91×10^{-2}
rs2075799 *HSPA1L* *(T/C)*	T	*LY6G5C*	↓(-16.8858)	5.70×10^{-64}	0
		HSPA1L	↓(-15.7141)	1.21×10^{-55}	0
		STK19P	↑(14.1055)	3.52×10^{-45}	0
		HLA-DRB1	↓(-11.786)	4.61×10^{-32}	0
		LINC00243	↓(-10.4831)	1.03×10^{-25}	0
		LSM2	↓(-10.4307)	1.79×10^{-25}	0
		DDAH2	↓(-9.8032)	1.09×10^{-22}	0
		HLA-DRB5	↓(-9.3342)	1.02×10^{-20}	0
		HLA-DQA2	↑(8.4204)	3.76×10^{-17}	0
		HLA-DRB6	↓(-7.7183)	1.18×10^{-14}	0
		HCG27	↑(7.3084)	2.71×10^{-13}	0
		SKIV2L	↑(7.3042)	2.79×10^{-13}	0
		LY6G5B	↓(-6.5791)	4.73×10^{-11}	0
		HLA-DQB1	↓(-6.4458)	1.15×10^{-10}	0
		DDR1	↓(-6.2233)	4.86×10^{-10}	0
		HLA-C	↓(-5.997)	2.01×10^{-9}	1.96×10^{-5}
		RNF5	↑(5.521)	3.37×10^{-8}	1.34×10^{-4}
		HLA-DQB1-AS1	↓(-5.3978)	6.74×10^{-8}	2.53×10^{-4}
		FKBPL	↑(5.3441)	9.09×10^{-8}	3.09×10^{-4}
		PSORS1C3	↓(-5.2372)	1.63×10^{-7}	5.01×10^{-4}
		CSNK2B	↑(5.2291)	1.70×10^{-7}	5.26×10^{-4}
		PPT2	↑(5.2252)	1.74×10^{-7}	5.38×10^{-4}
		CYP21A1P	↑(4.8044)	1.55×10^{-6}	4.33×10^{-3}
		POU5F1	↓(-4.7942)	1.63×10^{-6}	4.48×10^{-3}
		C4A	↑(4.698)	2.63×10^{-6}	6.98×10^{-3}
		MICB	↑(4.4784)	7.53×10^{-6}	1.98×10^{-2}
		TNXA	↑(4.4345)	9.23×10^{-6}	2.40×10^{-2}
		PBX2	↓(-4.2738)	1.92×10^{-5}	4.78×10^{-2}
rs4616886 *HSPA4* (T/C)	C	*SHROOM1*	↓(-28.95)	2.79×10^{-184}	0
		HSPA4	↑(9.9081)	3.84×10^{-23}	0
		UQCRQ	↓(-7.4652)	8.30×10^{-14}	0
		SLC22A5	↑(5.6741)	1.40×10^{-8}	7.06×10^{-5}
		LEAP2	↓(-4.9333)	8.08×10^{-7}	2.42×10^{-3}
rs13161158 *HSPA4* (T/C)	C	*AFF4*	↑(7.2197)	5.21×10^{-13}	0
		HSPA4	↑(6.2315)	4.62×10^{-10}	0
		SLC22A5	↑(6.2054)	5.46×10^{-10}	0
		LEAP2	↓(-4.494)	6.98×10^{-6}	1.84×10^{-2}

		SLC22A4	↑(4.2745)	1.92×10^{-5}	4.77×10^{-2}
rs753856 *HSPA6* *(G/C)*	G	*FCGR2B*	↓(-15.4623)	6.22×10^{-54}	0
		PSMD1	↑(10.4986)	8.77×10^{-26}	0
		FCGR2A	↑(6.2304)	4.65×10^{-10}	0
		FCRLB	↓(-5.9836)	2.18×10^{-9}	1.96×10^{-5}
		RP11-122G18.5	↓(-4.6411)	3.46×10^{-6}	9.25×10^{-3}
		FCGR2C	↑(4.3926)	1.12×10^{-5}	2.91×10^{-2}
rs706121 *BAG1* *(C/T)*	C	*AQP3*	↑(10.9221)	9.05×10^{-28}	0
		NOL6	↑(8.3209)	8.74×10^{-17}	0
		NFX1	↑(7.9588)	1.74×10^{-15}	0
		BAG1	↓(-7.1188)	1.09×10^{-12}	0
		MCMBP	↓(-24.3941)	1.97×10^{-131}	0
rs7749960 *BAG2* *(A/C)*	A	*RAB23*	↓(-12.1297)	7.34×10^{-34}	0
		KIAA1586	↓(-10.1783)	2.48×10^{-24}	0
		BAG2	↓(-6.5795)	4.72×10^{-11}	0
		ZNF451	↓(-5.1429)	2.70×10^{-7}	8.46×10^{-4}
rs2234962 *BAG3* *(C/T)*	C	*MCMBP*	↓(-24.3941)	1.97×10^{-131}	0
		BAG3	↑(5.7044)	1.17×10^{-8}	6.43×10^{-5}
rs196329 *BAG3* *(A/G)*	A	*BAG3*	↓(-11.8884)	1.36×10^{-32}	0
		MCMBP	↑(11.8464)	2.25×10^{-32}	0
		KRIT1	↑(11.8166)	3.20×10^{-32}	0
		CYP51A1	↑(9.3365)	9.97×10^{-21}	0
		ANKIB1	↓(-6.0778)	1.22×10^{-9}	1.31×10^{-5}
		AKAP9	↑(4.5979)	4.27×10^{-6}	1.14×10^{-2}
Effect on the level of gene expression: decrease (↓)/increase (↑)					

Table S9. The impact of SNPs in genes encoding HSP70, gene expression (cis-eQTL) in blood vessels, and brain and blood cells (according to the GTEx Portal browser, https://gtexportal.org) (accessed on 10 May 2026).

SNP	Gene Expressed	P-Value	Effect (NES)	Tissue
rs2227956 *HSPA1L* (G/**A**)	*HLA-DRB5*	1.40×10^{-27}	↓(-0.48)	Whole Blood
	HLA-DRB5	1.90×10^{-23}	↓(-0.56)	Artery - Tibial
	MICB	1.80×10^{-21}	↑(0.34)	Whole Blood
	HLA-DRB5	3.00×10^{-18}	↓(-0.65)	Artery - Aorta
	HLA-DRB9	4.60×10^{-18}	↑(0.48)	Whole Blood
	HLA-DRB6	7.90×10^{-16}	↑(0.48)	Whole Blood
	HLA-DRB5	1.70×10^{-14}	↓(-0.80)	Brain - Cerebellar Hemisphere
	HLA-DRB6	2.60×10^{-14}	↑(0.42)	Artery - Tibial
	HLA-DRB5	6.90×10^{-14}	↓(-0.80)	Brain - Cortex
	HLA-DRB5	1.40×10^{-13}	↓(-0.70)	Brain - Nucleus accumbens (basal ganglia)

	HLA-DRB5	3.40×10^{-13}	↓(-0.63)	Brain - Caudate (basal ganglia)
	HLA-DRB5	3.50×10^{-13}	↓(-0.76)	Brain - Putamen (basal ganglia)
	HLA-DRB5	3.10×10^{-12}	↓(-0.68)	Brain - Hippocampus
	HLA-DRB5	6.80×10^{-12}	↓(-0.65)	Brain - Hypothalamus
	LY6G5B	2.10×10^{-11}	↓(-0.15)	Artery - Tibial
	HLA-DRB5	6.80×10^{-11}	↓(-0.66)	Brain - Frontal Cortex (BA9)
	HLA-DRB6	1.50×10^{-10}	↑(0.47)	Artery - Aorta
	HLA-DRB5	1.90×10^{-10}	↓(-0.64)	Artery - Coronary
	HLA-DRB5	2.60×10^{-10}	↓(-0.65)	Brain - Cerebellum
	HLA-DQB2	3.20×10^{-10}	↑(0.35)	Whole Blood
	LY6G5B	1.20×10^{-9}	↓(-0.27)	Artery - Coronary
	LY6G5B	1.90×10^{-9}	↓(-0.19)	Artery - Aorta
	C4B	1.90×10^{-9}	↑(0.39)	Whole Blood
	HLA-DQB1	2.00×10^{-9}	↓(-0.27)	Whole Blood
	HLA-B	2.20×10^{-9}	↓(-0.17)	Whole Blood
	HLA-DRB5	2.60×10^{-9}	↓(-0.64)	Brain - Substantia nigra
	HSPA1L	2.70×10^{-9}	↓(-0.45)	Brain - Cerebellar Hemisphere
	HLA-C	3.00×10^{-9}	↓(-0.42)	Artery - Aorta
	HLA-DRB5	3.40×10^{-9}	↓(-0.61)	Brain - Amygdala
	HCG27	3.80×10^{-9}	↑(0.22)	Artery - Tibial
	CYP21A2	4.00×10^{-9}	↑(0.56)	Brain - Hippocampus
	HCG27	4.10×10^{-9}	↑(0.40)	Brain - Cerebellar Hemisphere
	HLA-C	4.10×10^{-9}	↓(-0.23)	Artery - Tibial
	HCG27	4.10×10^{-9}	↑(0.44)	Brain - Nucleus accumbens (basal ganglia)
	LY6G5B	6.00×10^{-9}	↓(-0.28)	Brain - Hippocampus
	HLA-DQB2	6.00×10^{-9}	↑(0.44)	Artery - Aorta
	LY6G5B	6.70×10^{-9}	↓(-0.32)	Brain - Cerebellum
	HLA-DRB5	9.40×10^{-9}	↓(-0.58)	Brain - Anterior cingulate cortex (BA24)
	ZBTB12	1.10×10^{-8}	↓(-0.30)	Whole Blood
	HLA-DQB2	1.30×10^{-8}	↑(0.33)	Artery - Tibial
	CYP21A2	1.30×10^{-8}	↑(0.64)	Brain - Cerebellar Hemisphere
	CYP21A2	2.00×10^{-8}	↑(0.53)	Brain - Hypothalamus

	HLA-DQA2	2.20×10^{-8}	↑(0.35)	Whole Blood
	CYP21A2	2.70×10^{-8}	↑(0.67)	Brain - Cerebellum
	CYP21A2	3.20×10^{-8}	↑(0.34)	Artery - Tibial
	LY6G5B	3.30×10^{-8}	↓(-0.30)	Brain - Substantia nigra
	HCG27	3.60×10^{-8}	↑(0.20)	Artery - Aorta
	HLA-DQB1	8.10×10^{-8}	↓(-0.34)	Artery - Aorta
	HCG27	9.70×10^{-8}	↑(0.13)	Whole Blood
	LY6G5B	1.30×10^{-7}	↓(-0.11)	Whole Blood
	CYP21A2	1.40×10^{-7}	↑(0.46)	Brain - Putamen (basal ganglia)
	HLA-DQB1-AS1	1.70×10^{-7}	↓(-0.20)	Whole Blood
	HLA-DQB1	1.70×10^{-7}	↓(-0.25)	Artery - Tibial
	ATF6B	1.90×10^{-7}	↑(0.31)	Brain - Caudate (basal ganglia)
	C4A	2.20×10^{-7}	↓(-0.31)	Whole Blood
	HCG27	2.80×10^{-7}	↑(0.40)	Brain - Caudate (basal ganglia)
	CYP21A2	3.10×10^{-7}	↑(0.44)	Brain - Caudate (basal ganglia)
	HLA-DRB6	3.60×10^{-7}	↑(0.47)	Artery - Coronary
	CYP21A2	3.80×10^{-7}	↑(0.40)	Brain - Nucleus accumbens (basal ganglia)
	CFB	4.20×10^{-7}	↓(-0.16)	Artery - Tibial
	BAG6	6.20×10^{-7}	↑(0.30)	Brain - Hypothalamus
	LY6G5B	7.30×10^{-7}	↓(-0.20)	Brain - Cerebellar Hemisphere
	LY6G5B	8.80×10^{-7}	↓(-0.21)	Brain - Nucleus accumbens (basal ganglia)
	CYP21A2	9.20×10^{-7}	↑(0.31)	Whole Blood
	HLA-DQA2	1.00×10^{-6}	↑(0.33)	Artery - Tibial
	MICB	1.00×10^{-6}	↑(0.41)	Brain - Putamen (basal ganglia)
	CYP21A2	1.10×10^{-6}	↑(0.45)	Brain - Anterior cingulate cortex (BA24)
	HLA-DRB1	1.30×10^{-6}	↓(-0.15)	Artery - Tibial
	HLA-DQB1-AS1	1.60×10^{-6}	↓(-0.21)	Artery - Tibial
	HSPA1L	1.70×10^{-6}	↓(-0.37)	Brain - Cerebellum
	C2	1.70×10^{-6}	↑(0.26)	Brain - Nucleus accumbens (basal ganglia)
	C4A	2.00×10^{-6}	↓(-0.22)	Artery - Tibial

	HLA-DRB1	2.30×10^{-6}	↓(-0.34)	Brain - Nucleus accumbens (basal ganglia)
	BAG6	2.70×10^{-6}	↑(0.18)	Artery - Tibial
	DDAH2	2.80×10^{-6}	↓(-0.09)	Whole Blood
	HLA-DRB9	3.40×10^{-6}	↑(0.35)	Artery - Aorta
	BAG6	4.10×10^{-6}	↑(0.40)	Brain - Anterior cingulate cortex (BA24)
	HLA-DRB6	4.50×10^{-6}	↑(0.51)	Brain - Hypothalamus
	HLA-DQB1-AS1	5.30×10^{-6}	↓(-0.26)	Artery - Aorta
	LY6G5B	6.30×10^{-6}	↓(-0.18)	Brain - Hypothalamus
	LY6G5B	6.40×10^{-6}	↓(-0.23)	Brain - Putamen (basal ganglia)
	ATF6B	7.00×10^{-6}	↑(0.16)	Artery - Tibial
	HCG27	7.10×10^{-6}	↑(0.45)	Brain - Amygdala
	HLA-DQA2	7.50×10^{-6}	↑(0.38)	Artery - Aorta
	HLA-DQB2	7.50×10^{-6}	↑(0.45)	Artery - Coronary
	HSPA1L	7.60×10^{-6}	↓(-0.34)	Brain - Nucleus accumbens (basal ganglia)
	HLA-DQB1	8.80×10^{-6}	↓(-0.42)	Brain - Cerebellar Hemisphere
	BAG6	9.60×10^{-6}	↑(0.30)	Brain - Hippocampus
	HLA-DRB6	1.30×10^{-5}	↑(0.43)	Brain - Caudate (basal ganglia)
	HLA-DRB1	1.50×10^{-5}	↓(-0.37)	Brain - Cortex
	HLA-DRB1	1.60×10^{-5}	↓(-0.26)	Brain - Hypothalamus
	HLA-C	1.60×10^{-5}	↓(-0.38)	Brain - Hippocampus
	BAG6	1.80×10^{-5}	↑(0.26)	Brain - Caudate (basal ganglia)
	CYP21A2	1.80×10^{-5}	↑(0.42)	Brain - Frontal Cortex (BA9)
	HLA-DRB6	2.10×10^{-5}	↑(0.43)	Brain - Anterior cingulate cortex (BA24)
	LY6G5B	2.10×10^{-5}	↓(-0.29)	Brain - Amygdala
	ENSG00000272501	2.50×10^{-5}	↓(-0.11)	Whole Blood
	HLA-DRB1	2.50×10^{-5}	↓(-0.34)	Brain - Cerebellar Hemisphere
	LY6G5B	3.00×10^{-5}	↓(-0.18)	Brain - Frontal Cortex (BA9)
	LY6G5B	3.20×10^{-5}	↓(-0.18)	Brain - Caudate (basal ganglia)
	HLA-DRB6	3.60×10^{-5}	↑(0.42)	Brain - Frontal Cortex (BA9)
	CYP21A2	3.80×10^{-5}	↑(0.32)	Artery - Aorta
	CYP21A2	4.00×10^{-5}	↑(0.40)	Brain - Cortex

	HCG27	4.4×10^{-5}	↑(0.33)	Brain - Anterior cingulate cortex (BA24)
	HLA-DRB1	4.50×10^{-5}	↓(-0.25)	Brain - Caudate (basal ganglia)
	C4A	4.70×10^{-5}	↓(-0.42)	Brain - Cerebellar Hemisphere
	HLA-DRB1	4.70×10^{-5}	↓(-0.34)	Brain - Putamen (basal ganglia)
	EHMT2	4.70×10^{-5}	↑(0.16)	Brain - Putamen (basal ganglia)
	LY6G5C	5.40×10^{-5}	↓(-0.18)	Whole Blood
	ENSG00000272501	5.90×10^{-5}	↓(-0.35)	Brain - Cortex
	ENSG00000272221	6.50×10^{-5}	↓(-0.27)	Artery - Tibial
	HLA-DRB9	6.90×10^{-5}	↑(0.38)	Artery - Coronary
	ATF6B	7.90×10^{-5}	↑(0.28)	Brain - Hippocampus
	ENSG00000272501	8.40×10^{-5}	↓(-0.32)	Brain - Caudate (basal ganglia)
	HLA-DQB1	8.60×10^{-5}	↓(-0.40)	Brain - Hypothalamus
	C4B	8.80×10^{-5}	↓(-0.18)	Artery - Tibial
	BAG6	8.80×10^{-5}	↑(0.24)	Brain - Cerebellar Hemisphere
	STK19	9.40×10^{-5}	↓(-0.15)	Artery - Tibial
	ENSG00000272501	1.00×10^{-4}	↓(-0.35)	Brain - Frontal Cortex (BA9)
	HCG27	1.10×10^{-4}	↑(0.33)	Brain - Cortex
	C4A	1.20×10^{-4}	↓(-0.31)	Brain - Cortex
	EHMT2	1.20×10^{-4}	↑(0.15)	Brain - Caudate (basal ganglia)
	BAG6	1.20×10^{-4}	↑(0.25)	Brain - Nucleus accumbens (basal ganglia)
	HCG27	1.30×10^{-4}	↑(0.28)	Brain - Cerebellum
	DDAH2	1.50×10^{-4}	↓(-0.16)	Brain - Cerebellar Hemisphere
	ATP6V1G2	1.60×10^{-4}	↑(0.18)	Whole Blood
	PSORS1C3	1.80×10^{-4}	↑(0.17)	Whole Blood
	BAG6	1.90×10^{-4}	↑(0.32)	Brain - Frontal Cortex (BA9)
	SLC44A4	2.30×10^{-4}	↓(-0.22)	Whole Blood
	HLA-C	3.10×10^{-4}	↓(-0.13)	Whole Blood
	HSPA1B	3.30×10^{-4}	↓(-0.18)	Artery - Tibial
rs2075799 *HSPA1L* (C/**T**)	*STK19B*	3.00×10^{-9}	↑(0.96)	Brain - Cerebellar Hemisphere
	LSM2	1.40×10^{-8}	↓(-0.22)	Artery - Tibial

	STK19B	7.10×10^{-8}	↑(0.63)	Artery - Aorta
	LY6G5C	7.40×10^{-8}	↓(-0.33)	Whole Blood
	STK19B	4.30×10^{-7}	↑(0.98)	Brain - Cerebellum
	STK19B	9.70×10^{-7}	↑(0.79)	Brain - Cortex
	STK19B	1.20×10^{-6}	↑(0.79)	Brain - Hippocampus
	STK19	1.60×10^{-6}	↓(-0.27)	Artery - Tibial
	LSM2	4.40×10^{-6}	↓(-0.23)	Artery - Aorta
	STK19	1.20×10^{-5}	↓(-0.18)	Whole Blood
	STK19B	2.90×10^{-5}	↑(0.59)	Artery - Coronary
	VARS2	3.50×10^{-5}	↓(-0.25)	Artery - Tibial
	STK19B	3.70×10^{-5}	↑(0.60)	Brain - Nucleus accumbens (basal ganglia)
	VARS2	1.20×10^{-4}	↓(-0.33)	Artery - Aorta
	HSPA1L	1.50×10^{-4}	↓(-0.41)	Brain - Nucleus accumbens (basal ganglia)
	HLA-S	3.00×10^{-4}	↑(0.33)	Whole Blood
	ATP6V1G2	3.30×10^{-4}	↑(0.27)	Artery - Tibial
rs4616886 *HSPA4* (T/**C**)	*SHROOM1*	2.40E-36	↓(-0.48)	Whole Blood
rs706121 *BAG1* (C/**T**)	*NFX1*	1.00×10^{-27}	↓(-0.25)	Whole Blood
	NFX1	7.60×10^{-7}	↓(-0.15)	Artery - Aorta
	AQP3	4.50×10^{-5}	↓(-0.42)	Brain - Cerebellar Hemisphere
	NFX1	1.20×10^{-4}	↓(-0.09)	Artery - Tibial
rs7749960 *BAG2* (C/**A**)	*LINC00680*	1.20×10^{-7}	↓(-0.37)	Whole Blood
	KIAA1586	6.10×10^{-6}	↓(-0.54)	Artery - Coronary
	KIAA1586	2.40×10^{-5}	↓(-0.35)	Brain - Cerebellum
	LINC00680	8.70×10^{-5}	↓(-0.56)	Brain - Putamen (basal ganglia)
	KIAA1586	9.00×10^{-5}	↓(-0.35)	Brain - Cortex
	KIAA1586	1.00×10^{-4}	↓(-0.25)	Brain - Cerebellar Hemisphere
	KIAA1586	1.70×10^{-4}	↓(-0.22)	Artery - Tibial
	LINC00680	2.20×10^{-4}	↓(-0.44)	Brain - Caudate (basal ganglia)
	LINC00680	2.40×10^{-4}	↓(-0.49)	Brain - Cerebellar Hemisphere
	LINC00680	2.50×10^{-4}	↓(-0.47)	Brain - Anterior cingulate cortex (BA24)

	LINC00680	3.30×10^{-4}	↓(-0.31)	Artery - Tibial
	LINC00680	4.30×10^{-4}	↓(-0.47)	Brain - Cerebellum
rs2234962 *BAG3* (T/**C**)	*INPP5F*	3.00×10^{-6}	↓(-0.22)	Brain - Cerebellum
Effect on the level of gene expression: decrease (↓)/increase (↑)				

Table S10. Baseline and clinical characteristics of the studied groups.

**Baseline and Clinical Characteristics**		**IS Patients**	**Controls**	***p*-Value**
		**(n = 768)**	**(n = 794)**	
Age, Me [Q1; Q3]		63 [55; 70]	59 [53; 66]	<0.001
Sex	Males, N (%)	428 (55.7%)	296 (37.3%)	<0.001
	Females, N (%)	340 (44.3%)	498 (62.7%)	
Smoking	Yes, N (%)	387 (50.4%)	197 (24.8%)	<0.001
	No, N (%)	381 (49.6%)	597 (75.2%)	
Low physical activity	Yes, N (%)	290 (37.8%)	ND	
	No, N (%)	478 (62.2%)	ND	
Low fruit/vegetable consumption	Yes, N (%)	393 (51.2%)	ND	
	No, N (%)	375 (48.8%)	ND	
Age at onset of stroke, Me [Q1; Q3]		61 [54; 70] (n = 754)	-	
Number of strokes including event in question	1, N (%)	677 (89.9%)	-	
	2, N (%)	69 (9.2%)	-	
	3, N (%)	7 (0.9%)	-	
Stroke localization	Right/left middle cerebral artery basin, N (%)	631 (84.8%)	-	
	Vertebrobasilar basin, N (%)	113 (15.2%)	-	

Area of lesion in stroke, mm^2, Me [Q1; Q3]	97 [25; 460] (n = 741)	-	
Total cholesterol, mmol/L, Me [Q1; Q3]	5.2 [4.4; 5.9] (n = 521)	ND	
Triglycerides, mmol/L, Me [Q1; Q3]	1.3 [1.1; 1.8] (n = 515)	ND	
Prothrombin time, seconds, Me [Q1; Q3]	10.7 [10.1; 11.7] (n = 734)	ND	
International normalized ratio, Me [Q1; Q3]	1 [0.94; 1.09] (n = 515)	ND	
Activated partial thromboplastin time, seconds, Me [Q1; Q3]	32.7 [29; 37] (n = 516)	ND	
Continuous variables are presented as median [first quartile; third quartile], and categorical variables as number (percentage). Between-group comparisons were performed using the Mann–Whitney U test for continuous variables and Pearson's chi-squared test for categorical variables. ND, no data; IS, ischemic stroke.			

Table S11. Regulatory potential of selected genetic variants

SNP ID (HGVS ID)	EUR freq	HaploReg V4.2 (1)								RegulomeDB (2)		atSNP (3)
		Promoter histone marks	Enhancer histone marks	DNAse	Proteins bound	Motifs changed	GRASP QTL hits	Selected eQTL hits	dbSNP func annot	probability	ranking	TF binding
rs1043618 *HSPA1A* (NC_000006.12:g.31815730G>C)	0.39			49 tissues	10 bound proteins		1 hit	37 hits	5'-UTR	0.554	1f	+
rs2227956 *HSPA1L* (NC_000006.12:g.31810495G>A)	0.83	SKIN			ERALPHA_A	Sin3Ak-20	11 hits	112 hits	missense	0.554	1f	+
rs2075799 *HSPA1L* (NC_000006.12:g.31810752C>T)	0.08	SKIN, CRVX	SKIN		ERALPHA_A	8 altered motifs	2 hits	19 hits	synonymous	0.554	1f	+
rs1063391 *HSPA2* (NC_000014.9:g.64541533C>T)	0.70				CTCF	CDP,LBP-1,Rad21		12 hits	synonymous	0.690	1b	+
rs12173082 *HSPA4* (NC_000005.10:g.133053995C>G)										0.554	1f	+
rs13161158 *HSPA4* (NC_000005.10:g.133071996T>C)	0.08		7 tissues	SKIN		CTCF,Rad21,SMC3		2 hits	intronic	0.708	1f	+
rs4616886 *HSPA4* (NC_000005.10:g.133088072T>C)	0.18					AhR,Foxa,Pax-4	1 hit	2 hits	intronic	0.553	1f	+
rs11749966 *HSPA4* (NC_000005.10:g.133088354T>C)	0.06					4 altered motifs	1 hit		intronic	0.554	1f	+

rs12009 *HSPA5* (NC_000009.12:g.125235024G>A)	0.56	HRT	17 tissues	25 tissues	8 bound proteins	6 altered motifs	3 hits	22 hits	3'-UTR	0.949	1b	+
rs430397 *HSPA5* (NC_000009.12:g.125238840C>T)	0.07	MUS, HRT	11 tissues	8 tissues		DMRT5,GATA,Hltf	1 hit	2 hits	intronic	0.554	1f	+
rs753856 *HSPA6* (NC_000001.11:g.161526344C>G)	0.12	BLD	FAT			Evi-1,Ik-2		2 hits	missense	0.554	1f	+
rs1042665 *HSPA9* (NC_000005.10:g.138566650T>C)	0.25			HRT, SKIN		Brachyury, Eomes		1 hit	synonymous	0.554	1f	+
rs17155992 *HSPA14* (NC_000010.11:g.14849407G>A)	0.06					GR,Pax-5,p300		2 hits	intronic	0.553	1f	+
rs706121 *BAG1* (NC_000009.12:g.33260634C>T)	0.83		LNG, BLD	CRVX, BLD, SKIN	JUND, MAFK		1 hit	30 hits	intronic	0.554	1f	+
rs3778515 *BAG2* (NC_000006.12:g.57177911T>G)	0.23	9 tissues	12 tissues	6 tissues			2 hits	49 hits	intronic	0.554	1f	+
rs7749960 *BAG2* (NC_000006.12:g.57180758C>A)										0.553	1f	+
rs196336 *BAG3* (NC_000010.11:g.119654724T>C)	0.65	14 tissues	18 tissues	22 tissues	5 bound proteins	5 altered motifs		6 hits	intronic	0.403	1b	+
rs196345 *BAG3* (NC_000010.11:g.119661410G>A)	0.37		12 tissues	BLD,MUS,SKIN		5 altered motifs	2 hits	2 hits	intronic	0.554	1f	+

rs196325 *BAG3* (NC_000010.11:g.119669316A>G)	0.60	MUS, GI	18 tissues	HRT,PLCNT		Mef2,TEF, Zfp691	1 hit	1 hit	intronic	0.115	1f	+
rs2234962 *BAG3* (NC_000010.11:g.119670121T>C)	0.22		16 tissues	6 tissues		4 altered motifs	1 hit	2 hits	missense	0.554	1f	+
rs196329 *BAG3* (NC_000010.11:g.119673041A>G)	0.8		9 tissues			Ets,Nkx3	1 hit	5 hits	intronic	0.554	1f	+
rs12682626 *BAG4* (NC_000008.11:g.38189251A>G)										0.554	1f	+
rs11625397 *BAG5* (NC_000014.9:g.103561220C>T)	0.65	18 tissues	13 tissues	BLD		Rhox11	4 hits	33 hits	intronic	0.553	1f	+

1 –analysis of regulatory potential SNPs using the HaploReg V4.2 resource (https://pubs.broadinstitute.org/mammals/haploreg/haploreg.php): we analysed Promoter histone marks, Enhancer histone marks; regions of hypersensitivity to DNase-1 (DNAse), bounding proteins; Motifs changed; GRASP QTL hits; Selected eQTL hits (expression or molecular QTL variants linked to trait-associated haplotype blocks);

2 – analysis of regulatory potential SNPs using the RegulomeDB resource (https://regulomedb.org/regulome-summary/): regulatory coefficients are presented - 1b (eQTL/caQTL + TF binding + any motif + Footprint + chromatin accessibility peak), 2b (TF binding + any motif + Footprint + chromatin accessibility peak), 1f (eQTL/caQTL + TF binding / chromatin accessibility peak)

3 - analysis of transcription factors binding using the atSNP resource (http://atsnp.biostat.wisc.edu/search): The effect of SNPs on DNA binding to transcription factors is presented (+/-)

N/D – no data available

Table S12. Sequence of forward (F), reverse (R), and extension (E) primers for SNPs genotyped using MassARRAY 4 technology

SNPs primers	Sequence		
rs2075799_F	5′	ACGTTGGATGTTATCAGGGCAGTCATCACG	3′
rs3778515_F	5′	ACGTTGGATGCGTTTCGATACTGAGACTGC	3′
rs4616886_F	5′	ACGTTGGATGCCCTCTTATTTTCCTGATGG	3′
rs196325_F	5′	ACGTTGGATGCCTTCCCCTTCCTGCAAATT	3′
rs7099687_F	5′	ACGTTGGATGATGGTTGAGGCTTTTGTGCG	3′
rs2234962_F	5′	ACGTTGGATGGCATGCCAGAAACCACTCAG	3′
rs1063391_F	5′	ACGTTGGATGTCGAGGATGGCATCTTCGAG	3′
rs12682626_F	5′	ACGTTGGATGAGACGTAAGCCACCATGCCA	3′
rs196345_F	5′	ACGTTGGATGAACTCTTGCAAAAACCCCTG	3′
rs12009_F	5′	ACGTTGGATGCCATTTAACCCTATTGCCCC	3′
rs12173082_F	5′	ACGTTGGATGCCCAGGGGCTCAATTCTAAG	3′
rs11749966_F	5′	ACGTTGGATGAGGGAGTTGGAGGAGTTTTG	3′
rs7749960_F	5′	ACGTTGGATGGGAAGACCTTAGGTCAAAAC	3′
rs430397_F	5′	ACGTTGGATGGCTTCATAGTAGACCGGAAC	3′
rs2227956_F	5′	ACGTTGGATGTGATCTTGTTCACCTTGCCG	3′
rs2075799_R	5′	ACGTTGGATGCAAGTCTGAGAAGGTACAGG	3′
rs3778515_R	5′	ACGTTGGATGTAGGAAGACAAAACCGGAGC	3′
rs4616886_R	5′	ACGTTGGATGTGTACTGCAGTGTACAGGTC	3′
rs196325_R	5′	ACGTTGGATGATCTTTACAGAGAAGTGGGC	3′
rs7099687_R	5′	ACGTTGGATGTGCATTAGTCCTGCAGTGTC	3′
rs2234962_R	5′	ACGTTGGATGTCCTTACCTCAGGTCCGTG	3′
rs1063391_R	5′	ACGTTGGATGTCACCATGCGGTTGTCGAAG	3′
rs12682626_R	5′	ACGTTGGATGGCATCCATTAGTTGTTACTC	3′
rs196345_R	5′	ACGTTGGATGTGTGACCATGGACAGGACTC	3′
rs12009_R	5′	ACGTTGGATGAGCAGAATACTGGTCTCAGG	3′
rs12173082_R	5′	ACGTTGGATGTCTAGTTTCGCTGAAACCTG	3′
rs11749966_R	5′	ACGTTGGATGACCACCAACTATCTCCACTG	3′
rs7749960_R	5′	ACGTTGGATGGGATGTCTTGCACTAAAGAG	3′
rs430397_R	5′	ACGTTGGATGCTCTGAATTCAGGACATTGC	3′
rs2227956_R	5′	ACGTTGGATGGGAGTTCCTCAGATCGAGGT	3′
rs2075799_E	5′	cCATCACGCCCCCAGC	3′
rs3778515_E	5′	TGCATCCTCAGCTAGT	3′
rs4616886_E	5′	CTGATGGCTAGCGTGT	3′
rs196325_E	5′	CTGCCTCCTCAGGAGTA	3′
rs7099687_E	5′	CAGGGGCTCATTGTTTC	3′
rs2234962_E	5′	cctCTGCCACCTGTCCAC	3′
rs1063391_E	5′	ggttAAGTCCACGGCCGGCGA	3′
rs12682626_E	5′	gcccCCATGCCAGGCCTGAATT	3′
rs196345_E	5′	cccaaACTCCTTGCTACTGTTTC	3′
rs12009_E	5′	cccgtTCTCAGGCCAAGCCCAATA	3′
rs12173082_E	5′	gggtAAGAAATGTGACAAAAATTGA	3′
rs11749966_E	5′	cccaTTATTTCATCATGGTAAGATGT	3′
rs7749960_E	5′	caTTAGGTCAAAACTTCAAAAGCATA	3′
rs430397_E	5′	gggaGGAACAGATCCTAGAAAAAAGA	3′
rs2227956_E	5′	ctttgTCACCTTGCCGGTGCTCTTGTCC	3′

Table S13. Distribution of *HSP70* genotypes in healthy controls and their correspondence to the Hardy-Weinberg equilibrium.

SNP	Genotypes	Controls	H_o (H_e)[1]	P[2] (pbonf)	IS patients	H_o (H_e)[3]	P[4] (pbonf)
rs1043618 *HSPA1A*	G/G	327 (41.2%)	0.448 (0.463)	>0.05	315 (41.9%)	0.465 (0.454)	>0.05
	G/C	355 (44.8%)			349 (46.5%)		
	C/C	111 (14.0%)			87 (11.6%)		
rs2227956 *HSPA1L*	A/A	435 (65.4%)	0.310 (0.309)	>0.05	465 (71.5%)	0.272 (0.253)	>0.05
	A/G	206 (31.0%)			177 (27.2%)		
	G/G	24 (3.6%)			8 (1.3%)		
rs2075799 *HSPA1L*	C/C	625 (79.6%)	0.190 (0.194)	>0.05	576 (76.6%)	0.215 (0.221)	>0.05
	C/T	149 (19.0%)			162 (21.5%)		
	T/T	11 (1.4%)			14 (1.9%)		
rs1063391 *HSPA2*	T/T	340 (43.9%)	0.458 (0.444)	>0.05	343 (45.6%)	0.450 (0.435)	>0.05
	T/C	355 (45.9%)			338 (45.0%)		
	C/C	79 (10.2%)			71 (9.4%)		
rs12173082 *HSPA4*	C/C	466 (59.4%)	0.344 (0.359)	>0.05	460 (60.8%)	0.340 (0.345)	>0.05
	C/G	269 (34.3%)			257 (34.0%)		
	G/G	49 (6.3%)			39 (5.2%)		
rs13161158 *HSPA4*	T/T	697 (87.7%)	0.120 (0.117)	>0.05	657 (86.0%)	0.134 (0.136)	>0.05
	T/C	95 (12.0%)			102 (13.3%)		
	C/C	2 (0.3%)			5 (0.7%)		
rs4616886 *HSPA4*	T/T	568 (75.2%)	0.222 (0.235)	>0.05	531 (75.3%)	0.211 (0.242)	0.002 (0,036)
	T/C	168 (22.3%)			149 (21.2%)		
	C/C	19 (2.5%)			25 (3.5%)		
rs11749966 *HSPA4*	T/T	722 (91.4%)	0.085 (0.083)	>0.05	685 (90.0%)	0.096 (0.098)	>0.05

	T/C	67 (8.5%)			73 (9.6%)		
	C/C	1 (0.1%)			3 (0.4%)		
rs12009 *HSPA5*	A/A	272 (34.4%)	0.470 (0.488)	>0.05	237 (31.1%)	0.509 (0.492)	>0.05
	A/G	371 (46.9%)			388 (50.8%)		
	G/G	148 (18.7%)			138 (18.1%)		
rs430397 *HSPA5*	C/C	657 (83.2%)	0.156 (0.164)	>0.05	637 (84.5%)	0.142 (0.154)	0.034 (0,776)
	C/T	123 (15.6%)			107 (14.2%)		
	T/T	10 (1.2%)			10 (1.3%)		
rs753856 *HSPA6*	C/C	611 (77.7%)	0.207 (0.210)	>0.05	619 (81.2%)	0.186 (0.171)	0.0096 (0,22)
	C/G	163 (20.8%)			142 (18.7%)		
	G/G	12 (1.5%)			1 (0.1%)		
rs1042665 *HSPA9*	T/T	482 (61.0%)	0.344 (0.341)	>0.05	443 (58.8%)	0.375 (0.348)	0.046 (1,061)
	T/C	272 (34.4%)			282 (37.5%)		
	C/C	36 (4.6%)			28 (3.7%)		
rs17155992 *HSPA14*	G/G	676 (85.9%)	0.136 (0.135)	>0.05	646 (84.7%)	0.143 (0.150)	>0.05
	G/A	107 (13.6%)			109 (14.3%)		
	A/A	4 (0.5%)			8 (1.0%)		
rs706121 *BAG1*	T/T	552 (73.3%)	0.230 (0.258)	0.004 (0,099)	538 (70.3%)	0.280 (0.265)	>0.05
	T/C	173 (23.0%)			214 (28.0%)		
	C/C	28 (3.7%)			13 (1.7%)		
rs3778515 *BAG2*	T/T	423 (57.2%)	0.377 (0.365)	>0.05	403 (57.3%)	0.367 (0.368)	>0.05
	T/G	279 (37.7%)			258 (36.7%)		
	G/G	38 (5.1%)			42 (6.0%)		
rs7749960 *BAG2*	C/C	594 (75.5%)	0.230 (0.228)	>0.05	623 (82.3%)	0.166 (0.170)	>0.05
	C/A	180 (22.9%)			126 (16.6%)		

	A/A	13 (1.6%)			8 (1.1%)		
rs196336 *BAG3*	C/C	270 (34.1%)	0.482 (0.487)	>0.05	244 (32.2%)	0.522 (0.486)	0.044 (1,009)
	C/T	382 (48.2%)			396 (52.2%)		
	T/T	140 (17.7%)			118 (15.6%)		
rs196345 *BAG3*	G/G	324 (43.2%)	0.435 (0.456)	>0.05	288 (42.5%)	0.431 (0.461)	>0.05
	G/A	326 (43.5%)			292 (43.1%)		
	A/A	100 (13.3%)			98 (14.4%)		
rs7099687 *BAG3*	A/A	487 (61.6%)	0.319 (0.348)	<0.05	461 (60.2%)	0.350 (0.347)	>0.05
	A/G	252 (31.9%)			268 (35.0%)		
	G/G	51 (6.5%)			37 (4.8%)		
rs196325 *BAG3*	G/G	267 (33.8%)	0.484 (0.487)	>0.05	257 (33.7%)	0.486 (0.487)	>0.05
	G/A	383 (48.5%)			371 (48.6%)		
	A/A	140 (17.7%)			135 (17.7%)		
rs2234962 *BAG3*	T/T	511 (64.8%)	0.301 (0.322)	>0.05	470 (62.0%)	0.336 (0.334)	>0.05
	T/C	238 (30.2%)			255 (33.6%)		
	C/C	40 (5.0%)			33 (4.4%)		
rs196329 *BAG3*	G/G	464 (58.4%)	0.355 (0.363)	>0.05	416 (55.8%)	0.364 (0.385)	>0.05
	G/A	282 (35.5%)			271 (36.4%)		
	A/A	48 (6.1%)			58 (7.8%)		
rs12682626 *BAG4*	A/A	477 (60.4%)	0.354 (0.342)	>0.05	458 (60.3%)	0.358 (0.340)	>0.05
	A/G	280 (35.4%)			272 (35.9%)		
	G/G	33 (4.2%)			29 (3.8%)		
rs11625397 *BAG5*	T/T	332 (44.6%)	0.467 (0.436)	>0.05	303 (43.0%)	0.474 (0.44)	>0.05
	T/C	347 (46.6%)			334 (47.4%)		
	C/C	66 (8.8%)			68 (9.6%)		

[1] – observed (Ho) and expected (He) heterozygosity in healthy controls; [2] – P-HWE in healthy controls; [3] – observed (Ho) and expected (He) heterozygosity in IS patients; 4 – P-HWE in IS patients
